# Spatial and temporal patterns of disease burden attributable to high BMI in Belt and Road Initiative countries, 1990–2019

**DOI:** 10.1017/S1368980024001253

**Published:** 2024-06-05

**Authors:** Yaxin Xu, Qizhe Wang, Tao Yu, Yan Han, Wei Dai, Sunfang Jiang, Xiaopan Li

**Affiliations:** 1 Department of Health Management Centre, Zhongshan Hospital, Fudan University, Shanghai, 200032, People’s Republic of China; 2 Department of General Practice, Zhongshan Hospital, Fudan University, Shanghai, 200032, People’s Republic of China; 3 School of Public Health, Fudan University, Shanghai, 200032, People’s Republic of China

**Keywords:** Belt and Road Initiative countries, Burden of disease, High BMI, Disability-adjusted life years, Average annual percent change, Trend analysis

## Abstract

**Objective:**

This study aimed to analyse the spatial and temporal patterns of disease burden attributed to high BMI (DB-hBMI) from 1990 to 2019 in Belt and Road Initiative (BRI) countries, in light of increasing hBMI prevalence worldwide.

**Design:**

The study was a secondary analysis of Global Burden of Disease 2019 (GBD 2019) that analysed (using Joinpoint regression analysis) numbers and the age-standardised rate of mortality and disability-adjusted life years (DALY) of hBMI-induced diseases and their trends from 1990 to 2019 and in the final decade.

**Setting:**

GBD 2019 study data for BRI countries were categorised by country, age, gender and disease.

**Participants:**

GBD 2019 data were used to analyse DB-hBMI in BRI countries.

**Results:**

In 2019, China, India and Russia reported the highest mortality and DALY among BRI countries. From 1990 to 2019, the age-standardised DALY increased in Southeast Asia and South Asia, whereas many European countries saw declines. Notably, Bangladesh, Nepal and Vietnam showed the steepest increases, with average annual percentage change (AAPC) values of 4·42 %, 4·19 % and 4·28 %, respectively (all *P* < 0·05). In contrast, Israel, Slovenia and Poland experienced significant reductions, with AAPC values of –1·70 %, –1·63 % and –1·58 %, respectively (all *P* < 0·05). The most rapid increases among males were seen in Vietnam, Nepal and Bangladesh, while Jordan, Poland and Slovenia recorded the fastest declines among females. Across most BRI countries, the burden of diabetes and kidney diseases related to hBMI showed a significant uptrend.

**Conclusion:**

DB-hBMI varies significantly by region, age, gender and disease type across BRI countries. It can pose a substantial threat to public health.

China’s Belt and Road Initiative (BRI) extends beyond geographical and political boundaries to enhance connections between countries in Asia, Europe and Africa^(1)^. While BRI countries share huge economic opportunities and potential, they also share some common publish health threats, such as obesity and related disease burdens^(2,3)^.

Obesity, a condition influenced by genetics, environment and lifestyle factors, significantly elevates the risk of chronic diseases, including type 2 diabetes, heart diseases, certain cancers, and bone and joint disorders^(4–6)^. High BMI (hBMI), defined as a BMI greater than 25 kg/m^2^ (which signifies overweight and obesity), has rapidly emerged as a global health crisis over the past few decades^(7)^. This global health challenge extends across borders, impacting populations worldwide.

Worldwide studies have highlighted the link between hBMI and higher rates of sickness and death^(8,9)^. In BRI countries, an alarming increase in obesity rates is seen, influenced by diverse cultural, dietary and socio-economic landscapes^(7,10)^. Moreover, the simultaneous occurrence of undernutrition and obesity, known as the ‘double burden of malnutrition’, presents a unique challenge in these regions^(11)^. This paradox highlights the multifaceted nature of nutritional issues faced by BRI countries.

While considering the intricate relationship between hBMI and disease burden in BRI countries, this study sought to analyse the disease burden attributable to hBMI (DB-hBMI) in BRI countries from 1990 to 2019. Knowledge of these dynamics is crucial for public health policymakers, healthcare providers and researchers, as it could inform evidence-based interventions and policies to mitigate health risks associated with obesity.

## Methods

### Data sources and definitions

This study was conducted using Global Burden of Disease 2019 (GBD 2019) study data obtained from the Institute for Health Metrics and Evaluation (IHME) website. All data for this study were obtained from the IHME website (https://www.healthdata.org/data-tools-practices/data-sources). The detailed methodology has been published elsewhere^(12,13)^.

Mortality, years lived with disability (YLD), years of life lost (YLL) and disability-adjusted life years (DALY) were recorded in this study. Age-standardised rates for mortality, YLD, YLL and DALY were calculated according to global age structure from 2019. The age-standardised rates were corrected by direct method and the world standard population in order to account for differences in population age structure. Our study followed the Guidelines for Accurate and Transparent Health Estimates Reporting (GATHER) to ensure its transparency and replicability (online Supplementary Table S1).

Our study focused on the BRI region with sixty-six participating member states, as detailed elsewhere^(14)^. The selection of BRI countries aligns with the GBD’s categorisation of global regions and international political and economic organisations. Additionally, we adopted two other key regional concepts: the World Bank region and the Socio-demographic Index (SDI) region. The SDI, developed in 2015, provides a comprehensive measure of a country or region’s development status by considering socio-economic factors. This index addresses the limitations of other indicators that solely reflect economic and developmental disparities among countries^(15)^. The World Bank classifies global economies into four income groups: low, lower middle, upper middle and high. Typically, low- and middle-income countries are considered developing, while high-income countries are classified as developed^(16)^.

### Statistical analysis

To quantify the DB-hBMI, we calculated age-standardised mortality, YLD, YLL and DALY for the BRI countries, stratified by gender, age and disease categories. Age-standardised estimates were adjusted to account for variations in the age distribution of populations, enabling meaningful comparisons across member states. We categorised age using three age groups: 15–49 years, 50–74 years and ≥ 75 years. Results of mortality, YLD, YLL and DALY are presented in absolute numbers and age-standardised rates per 100 000 population, along with their respective 95 % uncertainty intervals (UI)^(17)^.

To assess trends in DB-hBMI from 1990 to 2019, we employed the average annual percentage change (AAPC), determined using the Joinpoint Regression Program (version 4.0.4, released May, 2013)^(18)^. Each estimated metric was accompanied by a 95 % CI, providing a measure of statistical uncertainty. Statistical significance was defined as *P* < 0·05^(19)^.

## Results

The absolute numbers of age-standardised mortality, YLD, YLL and DALY in 2019 attributed to hBMI within BRI countries are presented in Table 1. In 2019, China reported 764 698·05 cases (95 % UI: 333 163·43, 1 310 557·19) of deaths and 24·83 million cases (95 % UI: 11·79, 40·55) of DALY attributed to hBMI.

**Table 1:** The absolute number of mortalities, YLD, YLL and DALY attributed to hBMI in the BRI countries in 2019 (Numbers and 95 % uncertainty intervals)

	Mortality		YLD		YLL		DALY	
Countries	*n*	95 % UI	*n*	95 % UI	*n*	95 % UI	*n*	95 % UI
Global	5 019 360	3 223 363·53, 7 110 735·87	40881595·32	24508834·36, 60876504·10	119383762·2	79596109·62, 163875518·20	160265357·5	105969034·16, 218870439·29
SDI levels								
High SDI	901 712·4	573 461·92, 1 289 615·62	10122478·78	6 183 273·93, 15026786·57	16686601·34	11174552·43, 22544644·27	26809080·12	18213630·98, 36348663·17
High–middle SDI	1 376 628	877 166·34, 1 953 868·91	10150023·08	5 993 344·26, 15279099·81	29437622·11	19353854·52, 40659948·15	39587645·2	26141026·08, 54009606·73
Middle SDI	1 647 281	1 051 541·86, 2 333 137·27	12878701·91	7 720 777·62, 19263210·75	42587187·55	28028288·41, 58701677·59	55465889·46	36710764·42, 75810217·60
Low–middle SDI	804 748·2	490 929·88, 1 158 653·50	5 809 393·79	3 370 046·12, 8 900 243·99	22197728·4	13841015·51, 31191364·79	28007122·19	17469918·70, 39227161·94
Low SDI	285 467·8	162 714·06, 429 330·36	1 892 548·01	1 038 467·39, 2 966 649·54	8 384 282·2	4 913 688·72, 12264607·41	10276830·2	6 088 925·88, 14897026·50
World Bank income levels								
World Bank high income	1 129 489	717 022·29, 1 615 082·54	12122739·9	7 399 228·01, 18044607·41	20392869·11	13681655·56, 27577282·04	32515609·01	22039488·01, 44079689·44
World Bank upper middle income	2 031 572	1 233 150·71, 2 979 293·52	16184636·63	9 326 744·34, 24873884·73	47170541·45	29510961·20, 67439620·77	63355178·09	40093386·51, 89552347·54
World Bank lower middle income	1 669 245	1 086 415·68, 2 312 343·75	11395503·53	6 967 229·61, 16881143·26	46346137·12	30995385·44, 63110831·84	57741640·65	38606805·99, 78205714·84
World Bank low income	185 479·5	105 038·11, 280 056·57	1 149 894·67	639 316·73, 1 807 604·74	5 382 606·31	3 104 278·18, 7 936 760·36	6 532 500·97	3 837 450·86, 9 557 327·37
East Asia								
China	########	333 163·43, 1 310 557·19	6 510 941·31	3 083 112·91, 11058881·54	18319099·53	30510023·55, 24830040·84	24830040·84	11788975·74, 40545898·53
Central Asia								
Armenia	4463·21	2959·97, 6240·38	27 873·24	17 666·62, 40 410·59	95 240·40	128 725·91, 123 113·64	123 113·64	84 876·53, 164 797·14
Azerbaijan	14 333·01	9223·45, 19 680·47	75 219·13	46 891·19, 110 178·82	363 690·87	494 292·42, 438 910·00	438 910·00	293 225·69, 593 146·72
Georgia	8567·96	5519·09, 12 100·70	38 701·64	24 341·68, 56 287·53	173 252·07	238 906·06, 211 953·72	211 953·72	143 543·74, 286 655·30
Kazakhstan	22 827·82	15 045·94, 31 139·90	150 158·69	96 890·86, 213 937·70	553 272·98	746 472·36, 703 431·67	703 431·67	497 832·89, 927 382·70
Kyrgyzstan	4479·05	2793·69, 6450·32	24 732·29	15 209·14, 36 568·75	115 394·31	160 893·13, 140 126·60	140 126·60	92 915·24, 194 284·43
Mongolia	3587·19	2098·96, 5337·78	12 481·38	7606·87, 18 468·79	110 954·50	163 502·00, 123 435·89	123 435·89	75 315·71, 178 620·74
Tajikistan	4664·91	2471·86, 7338·73	25 960·95	14 161·60, 40 696·54	128 625·55	200 399·21, 154 586·49	154 586·49	85 771·98, 235 092·75
Turkmenistan	6872·24	4363·52, 9710·85	32 228·70	20 536·76, 46 176·75	189 614·55	263 560·55, 221 843·25	221 843·25	148 726·97, 300 074·53
Uzbekistan	39 316·78	25 080·74, 54 534·60	180 824·06	115 156·35, 259 026·04	1 114 001·31	1 533 237·08, 1 294 825·38	1 294 825·38	863 510·18, 1 750 811·30
South Asia								
Bangladesh	43 802·93	20 447·04, 73 149·96	340 251·30	171 650·52, 561 284·35	1 301 880·22	2 095 983·68, 1 642 131·52	1 642 131·52	857 456·29, 2 597 199·37
Bhutan	307·48	161·64, 480·54	2482·77	1421·54, 3811·27	8149·80	12 576·99, 10 632·58	10 632·58	6026·86, 15 900·92
India	########	341 683·75, 846 048·17	4 827 871·52	2 831 223·38, 7 331 446·69	16189972·68	23404901·37, 21017844·19	21017844·19	12727180·45, 29671449·31
Nepal	9144·73	4420·22, 14 940·66	77 979·31	40 458·34, 123 854·21	250 463·03	402 269·66, 328 442·34	328 442·34	173 959·80, 516 118·00
Pakistan	########	56 802·53, 153 679·14	600 428·34	332 474·30, 932 230·44	3 016 768·66	4 606 869·10, 3 617 197·00	3 617 197·00	2 090 693·61, 5 436 223·22
Maldives	109·96	59·14, 172·55	1603·81	909·34, 2452·44	3202·72	4774·29, 4806·53	4806·53	
Sri Lanka	15 935·90	8843·09, 24 990·29	171 687·76	95 793·21, 257 545·93	362 422·31	565 471·79, 534 110·08	534 110·08	
Southeast Asia								
Cambodia	5254·57	2525·39, 8866·85	39 309·82	18 150·19, 67 442·06	148 853·67	247 761·99, 188 163·49	188 163·49	93 724·42, 309 928·53
Indonesia	########	112 309·32, 272 996·00	1 196 991·13	720 884·19, 1 756 171·36	5 811 306·16	8 338 420·10, 7 008 297·29	7 008 297·29	4 470 008·57, 9 865 705·66
Laos	3528·84	1849·51, 5366·94	24 020·87	13 194·31, 36 930·80	108 226·22	164 146·61, 132 247·09	132 247·09	72 428·35, 195 894·35
Malaysia	19 105·03	11 616·16, 27 845·81	194 827·05	123 825·15, 278 615·47	517 055·96	741 489·84, 711 883·01	711 883·01	465 516·28, 993 156·27
Burma	31 197·40	16 303·88, 48 521·18	198 790·88	106 909·24, 309 890·01	915 657·36	1 402 511·33, 1 114 448·24	1 114 448·24	610 636·78, 1 666 872·20
Philippines	65 200·21	37 358·30, 95 326·11	386 556·97	219 368·75, 576 478·26	1 945 079·53	2 811 972·97, 2 331 636·50	2 331 636·50	1 391 216·62, 3 317 185·97
Thailand	43 872·58	24 367·01, 69 412·87	478 633·24	281 990·39, 726 328·32	1 142 279·10	1 785 790·28, 1 620 912·34	1 620 912·34	991 658·49, 2 418 563·97
Vietnam	37 980·53	17 910·55, 65 256·40	261 562·50	117 509·20, 443 908·36	985 802·71	1 672 072·00, 1 247 365·21	1 247 365·21	595 339·15, 2 095 761·65
High-income Asia Pacific								
Brunei	187·29	101·55, 285·46	2557·56	1364·75, 4106·57	5273·40	7851·49, 7830·96	7830·96	4423·20, 11 522·95
Singapore	1777·38	1001·16, 2668·95	36 424·06	21 063·45, 54 891·14	40 055·19	56 932·96, 76 479·25	76 479·25	47 104·42, 109 578·70
North Africa and Middle East								
Afghanistan	22 048·09	13 613·48, 32 635·06	108 823·13	67 800·92, 160 469·75	682 610·47	1 020 105·19, 791 433·60	791 433·60	500 584·75, 1 153 715·64
Bahrain	1126·95	767·90, 1503·63	18 465·20	11 931·59, 26 085·71	30 513·61	40 473·18, 48 978·81	48 978·81	35 007·29, 63 283·96
Egypt	########	83 823·59, 184 740·86	633 650·17	410 033·95, 916 343·20	3 583 715·25	5 089 023·38, 4 217 365·43	4 217 365·43	2 842 298·41, 5 775 491·54
Iran	61 415·08	43 342·06, 81 357·86	582 597·88	376 877·74, 826 857·99	1 406 859·13	1 817 253·28, 1 989 457·00	1 989 457·00	1 441 099·58, 2 561 405·68
Iraq	36 481·23	23 495·51, 51 145·91	261 872·54	167 171·35, 376 816·77	974 924·10	1 381 835·36, 1 236 796·63	1 236 796·63	841 165·94, 1 672 505·65
Jordan	7548·11	5303·12, 9857·36	78 149·50	51 585·64, 110 102·96	184 468·48	239 622·49, 262 617·97	262 617·97	190 314·45, 340 017·08
Kuwait	2316·51	1595·66, 3014·26	44 045·57	29 428·61, 61 010·72	64 611·72	83 443·92, 108 657·29	108 657·29	80 241·70, 137 858·51
Lebanon	6164·95	3950·62, 8492·61	48 424·62	30 222·97, 70 218·53	133 535·19	181 964·63, 181 959·81	181 959·81	122 434·44, 242 467·98
Oman	2416·72	1677·12, 3161·99	26 302·56	17 036·92, 37 533·75	66 723·75	87 112·01, 93 026·30	93 026·30	67 425·26, 119 734·19
Palestine	2815·67	1805·51, 3959·70	23 036·11	14 227·18, 33 460·54	72 268·80	98 502·43, 95 304·92	95 304·92	65 962·55, 127 229·67
Qatar	962·61	649·13, 1306·75	28 982·95	19 066·28, 40 285·98	28 411·82	39 348·33, 57 394·76	57 394·76	42 382·33, 74 978·11
Saudi Arabia	28 038·52	18 986·99, 37 000·77	290 906·48	195 678·51, 403 655·05	892 913·40	1 195 029·50, 1 183 819·88	1 183 819·88	845 228·87, 1 535 554·66
Syrian Arab Republic	16 053·71	9631·36, 24 011·73	102 104·73	63 864·13, 149 477·96	423 161·11	630 618·50, 525 265·85	525 265·85	332 188·15, 746 285·31
Turkey	80 117·91	50 253·04, 114 778·60	713 462·88	446 590·60, 1 041 038·19	1 667 604·17	2 344 485·72, 2 381 067·06	2 381 067·06	1 609 700·55, 3 206 363·48
United Arab Emirates	7622·57	5210·04, 10 334·73	98 110·40	66 380·41, 135 590·14	275 109·96	379 385·57, 373 220·36	373 220·36	272 001·78, 487 534·07
Yemen	11 780·35	6337·49, 19 074·88	64 341·61	35 581·51, 99 114·85	346 538·97	559 857·31, 410 880·59	410 880·59	228 750·66, 650 581·28
Israel	5134·26	3040·91, 7427·71	54 105·21	31 047·62, 83 803·78	83 727·08	117 508·00, 137 832·30	137 832·30	
Central Europe								
Albania	3119·74	1771·99, 4890·07	21 037·60	12 573·34, 32 032·64	60 890·43	94 100·50, 81 928·02	81 928·02	50 720·29, 121 076·57
Bosnia and Herzegovina	5845·16	3631·55, 8459·47	46 588·37	28 319·46, 68 851·71	115 407·29	166 818·02, 161 995·66	161 995·66	107 608·59, 225 464·06
Bulgaria	22 741·70	13 987·43, 32 959·61	93 586·73	57 589·47, 137 908·49	459 445·83	657 461·12, 553 032·56	553 032·56	366 222·08, 772 293·38
Croatia	7659·50	4642·90, 11 205·63	57 876·02	36 060·65, 84 398·16	131 667·87	189 789·86, 189 543·89	189 543·89	125 153·53, 261 698·93
Czechia	17 453·23	10 756·18, 25 049·05	182 785·02	115 412·55, 266 537·04	300 269·94	424 258·01, 483 054·97	483 054·97	326 404·84, 656 158·20
Hungary	20 489·59	13 428·82, 28 601·44	139 455·45	88 413·83, 203 187·10	379 007·22	522 943·52, 518 462·67	518 462·67	363 144·51, 698 750·50
Montenegro	1218·46	778·91, 1721·66	8220·78	5247·38, 11 923·12	25 328·04	34 446·86, 33 548·82	33 548·82	22 998·93, 44 631·55
Macedonia	4675·73	2957·17, 6678·19	29 253·70	18 063·55, 42 628·84	98 102·17	139 373·03, 127 355·88	127 355·88	86 767·61, 175 118·53
Poland	57 514·19	35 751·18, 80 008·25	494 078·46	311 598·07, 703 927·33	1 067 673·36	1 460 769·23, 1 561 751·82	1 561 751·82	1 058 385·20, 2 102 608·24
Romania	42 107·26	28 402·21, 58 166·69	233 598·11	151 896·32, 335 404·70	796 408·40	1 074 964·28, 1 030 006·51	1 030 006·51	732 868·09, 1 367 060·48
Serbia	19 160·86	11 645·68, 27 428·88	122 263·38	75 648·20, 178 318·38	366 452·32	518 561·73, 488 715·70	488 715·70	320 977·58, 666 842·70
Slovakia	8778·55	5525·45, 12 672·95	62 246·72	38 927·63, 90 058·16	167 724·22	243 127·87, 229 970·94	229 970·94	153 796·83, 317 478·25
Slovenia	2604·75	1576·28, 3873·45	25 155·15	15 781·18, 36 673·05	40 448·95	58 985·79, 65 604·10	65 604·10	43 097·10, 91 626·50
Eastern Europe								
Belarus	19 271·67	11 336·13, 28 420·28	82 251·65	50 538·67, 120 496·06	399 760·47	584 567·95, 482 012·11	482 012·11	303 184·03, 687 250·79
Estonia	3026·34	1887·18, 4308·64	14 269·70	8985·93, 20 594·03	48 212·35	68 064·42, 62 482·05	62 482·05	42 458·25, 84 970·24
Latvia	4365·93	2770·38, 6275·45	22 452·74	13 966·74, 32 569·89	78 309·53	109 973·23, 100 762·27	100 762·27	68 570·41, 139 321·56
Lithuania	5806·73	3515·30, 8376·72	28 271·80	17 061·07, 42 087·05	101 158·35	142 431·48, 129 430·15	129 430·15	83 345·01, 178 820·38
Moldova	6769·34	4403·72, 9460·36	35 672·98	22 313·45, 52 438·92	142 063·68	193 438·61, 177 736·66	177 736·66	121 238·99, 239 858·64
Russian Federation	########	181 342·97, 390 743·23	1 304 994·87	816 177·97, 1 879 125·33	6 041 881·25	8 251 349·57, 7 346 876·11	7 346 876·11	4 996 033·55, 9 880 900·57
Ukraine	########	75 556·85, 161 688·65	416 151·97	262 157·25, 600 340·27	2 484 521·23	3 427 283·26, 2 900 673·21	2 900 673·21	1 963 237·07, 3 932 560·85
Western Europe								
Cyprus	802·42	470·88, 1191·83	8715·50	4989·62, 13 585·94	14 546·90	21 105·25, 23 262·41	23 262·41	14 076·63, 33 600·71
Greece	13 079·97	7306·49, 19 659·05	93 876·55	53 840·69, 147 851·13	209 409·98	301 688·48, 303 286·53	303 286·53	182 982·32, 431 464·30

YLD, years lived with disability; YLL, years of life lost; DALY, disability-adjusted life years; hBMI, high BMI; BRI, Belt and Road Initiative; UI, uncertainty interval; SDI, Socio-demographic Index.

As for SDI levels, the regions with the highest mortality rates attributed to hBMI were the high SDI regions and the low SDI regions, with 901 712·38 (95 % UI: 573 461·92, 1 289 615·62) and 285 467·84 cases (95 % UI: 162 714·06, 429 330·36), respectively, while the country with the highest DALY was in the middle SDI region, at 55·46 million cases (95 % UI: 36·11, 75·81).

Interestingly, significant geographic disparities were observed in the mortality and DALY attributed to hBMI across member countries. In 2019, the three countries with the highest mortality and DALY attributed to hBMI were China, India and the Russian Federation. Based on DALY, the top three countries in Central Asia with the highest burden attributed to hBMI were Uzbekistan, Kazakhstan and Azerbaijan. In Central Europe, Poland, Romania and Bulgaria ranked highest. In Eastern Europe, the countries with the highest DALY were the Russian Federation, Ukraine and Belarus. In North Africa and the Middle East, Egypt, Turkey and Iran exhibited the highest DALY. Finally, in Southeast Asia, the leading countries were Indonesia, the Philippines and Thailand.

**Fig. 1:**
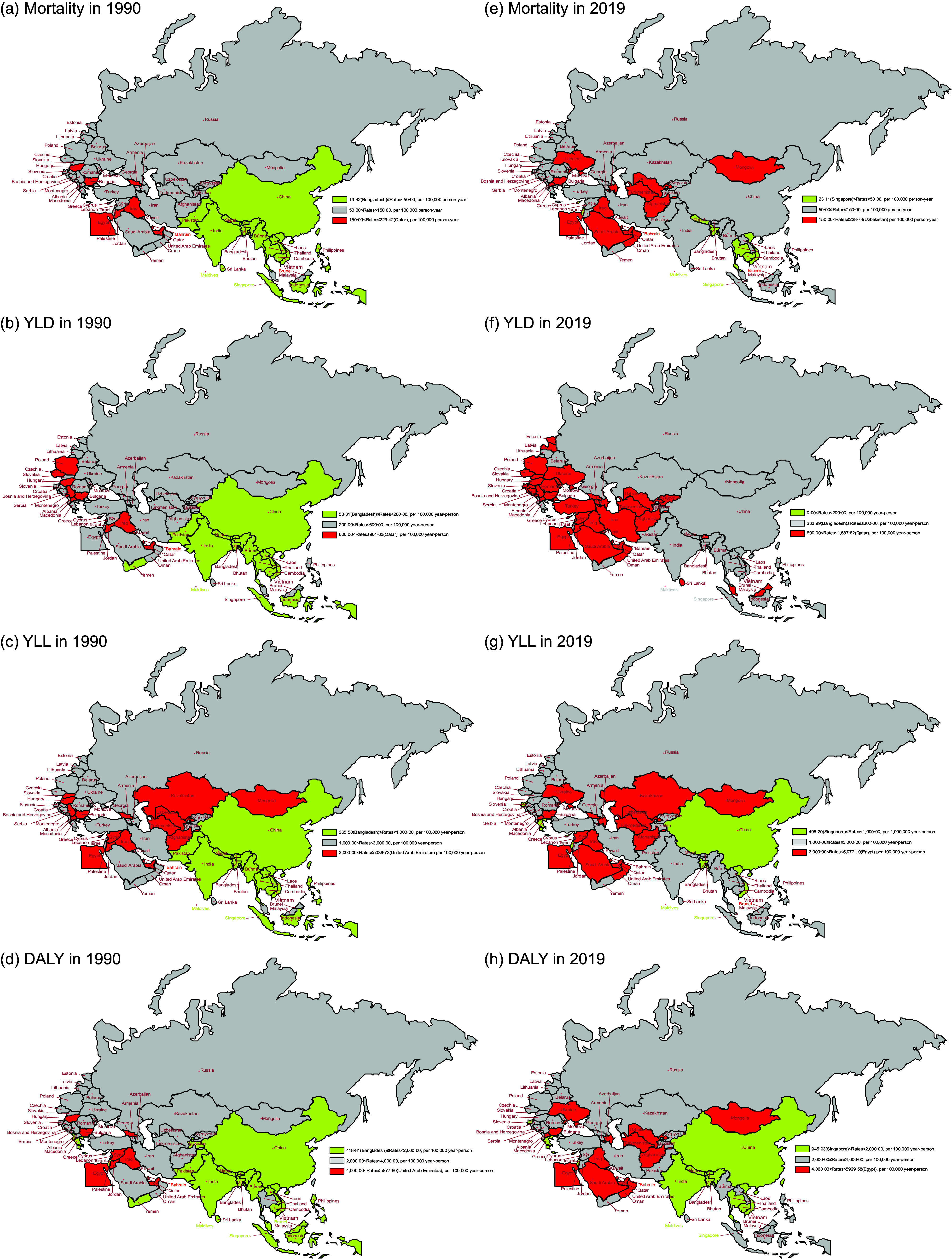
Age-standardised mortality, YLD, YLL and DALY attributed to hBMI for BRI countries in 1990 and 2019. (a) Age-standardised mortality in 1990. (b) Age-standardised YLD in 1990. (c) Age-standardised YLL in 1990. (d) Age-standardised DALY in 1990. (e) Age-standardised mortality in 2019. (f) Age-standardised YLD in 2019. (g) Age-standardised YLL in 2019. (h) Age-standardised DALY in 2019. YLD, years lived with disability; YLL, years of life lost; DALY, disability-adjusted life years; BRI, Belt and Road Initiative

Figure 1 shows the age-standardised mortality, YLD, YLL and DALY attributed to hBMI in BRI countries during 1990 and 2019. In 1990, regions with higher mortality, YLD, YLL and DALY attributed to hBMI were primarily concentrated in North Africa and the Middle East and Central Europe. In BRI countries, countries in North Africa and Middle East with the highest DALY attributed to hBMI were the United Arab Emirates (5877·80 per 100 000 population), Qatar (5514·94 per 100 000 population) and Iraq (5512·65 per 100 000 population). Conversely, the lowest DALY were reported by the United Arab Emirates (5877·80 per 100 000 population), Qatar (5514·94 per 100 000 population) and Iraq (5512·65 per 100 000 population) in Asia. In 2019, compared to 1990, most member countries of the BRI experienced either stable or increased DALY attributed to hBMI. Notably, higher rates were still concentrated in North Africa, the Middle East, Central Europe and Central Asia. For more detailed information, refer to online Supplementary Table S2.

**Fig. 2:**
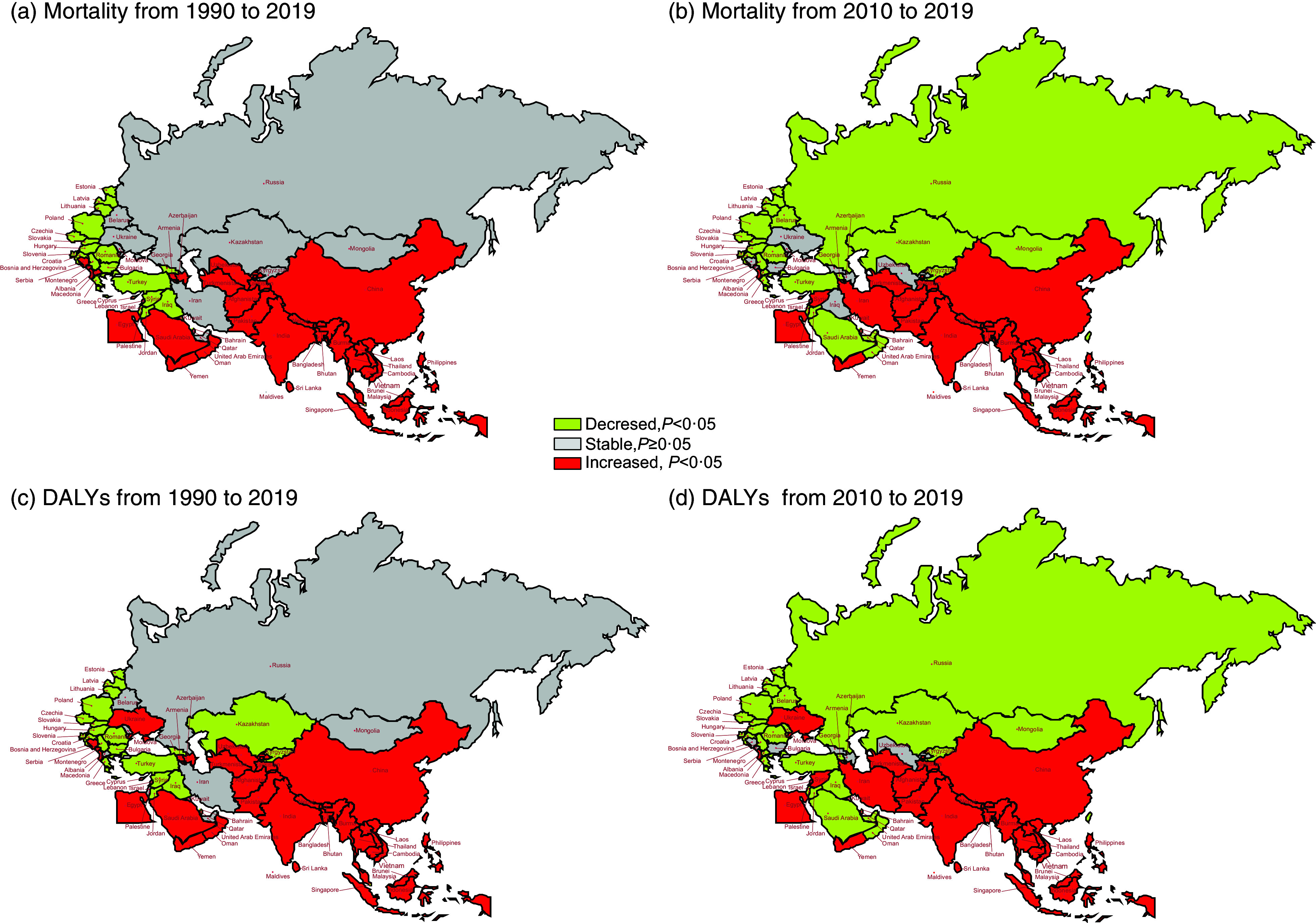
The temporal trend in the age-standardised mortality and DALY rate attributed to hBMI for 1990–2019 and 2010–2019 in BRI countries. (a) The AAPC of age-standardised mortality rate from 1990 to 2019. (b) The AAPC of age-standardised mortality rate from 2010 to 2019. (c) The AAPC of age-standardised DALY rate from 1990 to 2019. (d) The AAPC of age-standardised DALY rate from 2010 to 2019. DALY, disability-adjusted life years; hBMI, high BMI; BRI, Belt and Road Initiative; AAPC, average annual percentage change

Figure 2 illustrates the temporal trends of age-standardised mortality and DALY attributed to hBMI in BRI countries from 1990 to 2019 and 2010 to 2019. Between 1990 and 2019, there were distinctive trends in age-standardised mortality and DALY attributed to hBMI observed among the BRI countries. Notably, Southeast Asian and South Asian nations generally exhibited an upward trajectory in both mortality and DALY, whereas most European countries experienced a significant decline. In particular, South Asian countries, including Bangladesh and Nepal, along with the Southeast Asian nation Vietnam, demonstrated the most significant increases in DALY, with AAPC values of 4·42 % (95 % CI: 4·00 %, 4·83 %), 4·19 % (95 % CI: 3·97 %, 4·41 %) and 4·28 % (95 % CI: 3·92 %, 4·64 %), respectively.

Conversely, Israel, together with Slovenia and Poland, saw notable decreases in DALY, with APCC values of –1·70 % (95 % CI: –1·85 %, –1·55 %), –1·63 % (95 % CI: –1·77 %, –1·48 %) and –1·58 % (95 % CI: –1·71 %, –1·45 %), respectively. While some countries experienced a rising or stable trend in DALY from 1990 to 2019, a downward trend emerged in the period from 2010 to 2019 for nations such as Oman, Mongolia and the Russian Federation, with APCC values of –1·94 % (95 % CI: –2·33 %, –1·56 %), –1·09 % (95 % CI: –1·63 %, –0·55 %) and –2·25 % (95 % CI: –2·77 %, –1·72 %), respectively. See online Supplementary Table S3 for more details.

**Fig. 3:**
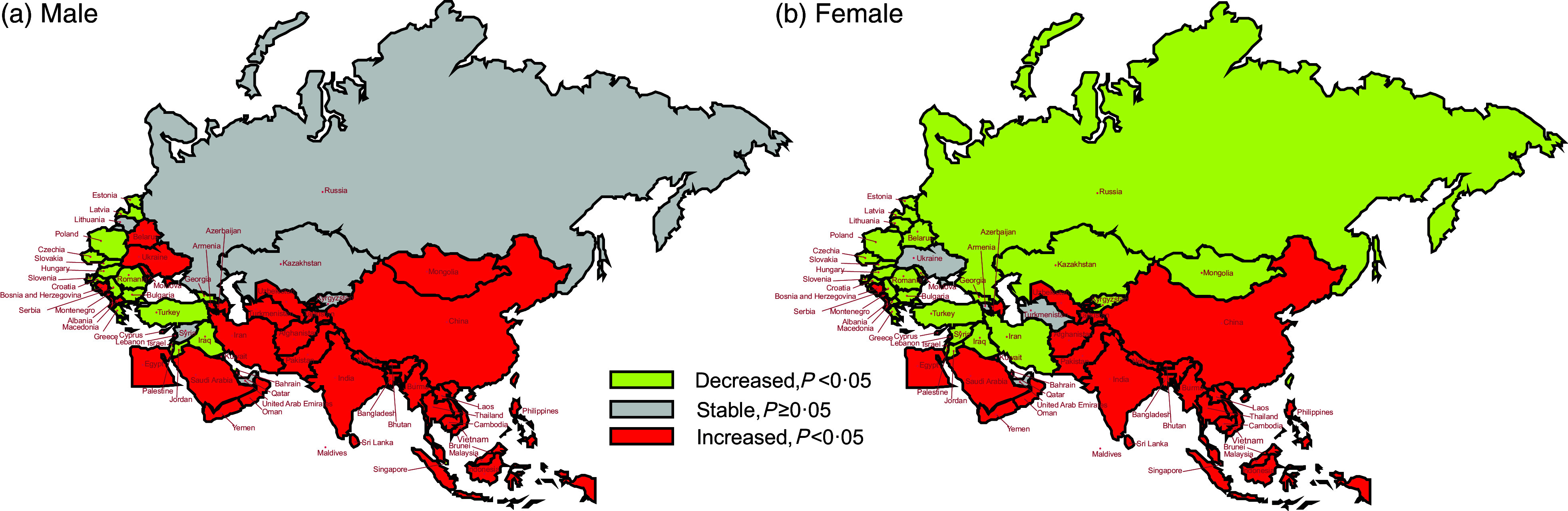
The temporal trend in the age-standardised DALY rate attributed to hBMI, stratified by gender for 1990–2019 in BRI countries. (a) The AAPC of age-standardised DALY rate in males. (b) The AAPC of age-standardised DALY rate in females. DALY, disability-adjusted life years; hBMI, high BMI; BRI, Belt and Road Initiative; AAPC, average annual percentage change

Figure 3 depicts the trend changes in age-standardised DALY for males and females in BRI countries. In South Asia and Southeast Asia, except for Maldives, both males and females witnessed an increasing trend in DALY (all *P* < 0·05). For males, most countries in East Asia, South Asia, Southeast Asia and Central Asia, except for Georgia, Kazakhstan and Kyrgyzstan, showed an upward trend in DALY (all *P* < 0·05).

Among these countries, Vietnam, Bangladesh and Nepal exhibited the most rapid increases, with AAPC values of 5·10 % (95 % CI: 4·73 %, 5·47 %), 4·79 % (95 % CI: 4·60 %, 4·97 %) and 4·61 % (95 % CI: 4·20 %, 5·01 %), respectively. Conversely, the most significant reductions were noted in Israel, Slovenia and Czechia, with their AAPC values being –1·75 % (95 % CI: –1·91 %, –1·58 %), –1·65 % (95 % CI: –1·78 %, –1·52 %) and –1·63 % (95 % CI: –1·83 %, –1·43 %), respectively. Unlike males, females in Central Asia (Mongolia and Kazakhstan), Southeast Asia (Maldives), North Africa and the Middle East (Iran, Kuwait, Lebanon, Palestine and Syrian Arab Republic), Central Europe (Macedonia) and Eastern Europe (Belarus, Moldova, Lithuania and Russian Federation) showed a decreasing trend in DALY (all *P* < 0·05).

In female populations, Jordan (North Africa and the Middle East) along with Poland and Slovenia (Central Europe) experienced the most notable declines, with their AAPC values being –0·18 % (95 % CI: –0·27 %, –0·08 %), –1·53 % (95 % CI: –1·67 %, –1·40 %) and –1·63 % (95 % CI: –1·83 %, –1·43 %), respectively (online Supplementary Table S4).

**Fig. 4:**
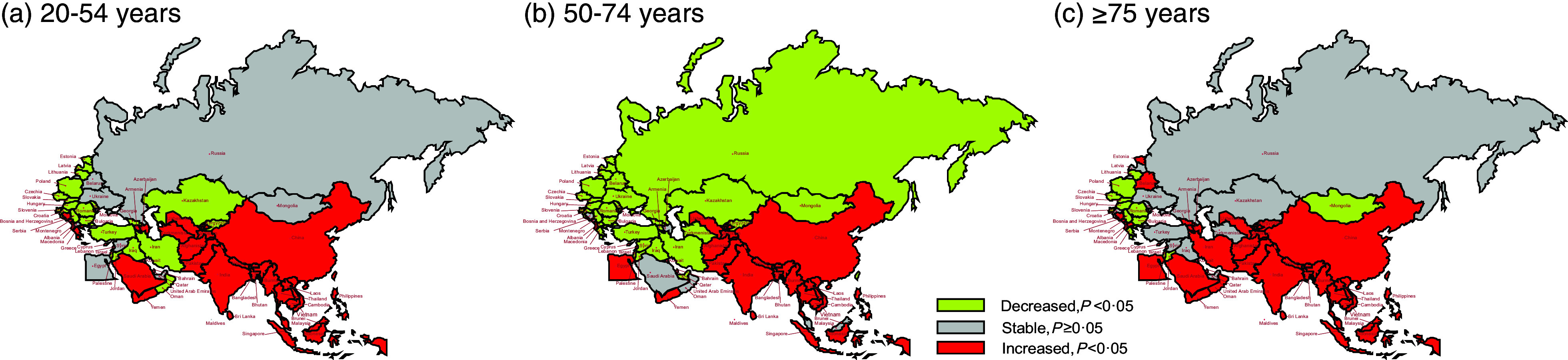
The temporal trend in the DALY rate attributed to hBMI, stratified by age for 1990–2019 in BRI countries. (a) The AAPC of DALY rate in people aged 20–54 years. (b) The AAPC of DALY rate in people aged 50–74 years. (c) The AAPC of DALY rate in people aged ≥75 years. DALY, disability-adjusted life years; hBMI, high BMI; BRI, Belt and Road Initiative; AAPC, average annual percentage change

Figure 4 depicts the trend changes in age-standardised DALY across different age groups within BRI countries. Over this period, there was a notable increase in DALY across all age groups in China, South Asia and Central Asia, except for Turkmenistan (all *P* < 0·05). Individuals aged 20–54 years in most countries in North Africa and the Middle East, Central Europe, Eastern Europe, and Western Europe saw declines in DALY. In the 50–74-year age group, most countries in Central Asia, North Africa and the Middle East, and Europe experienced a decrease in DALY. For those aged 75 years and above, Nepal, Vietnam and Uzbekistan exhibited the most significant increases in DALY, with AAPC values of 4·16 % (95 % CI: 3·97 %, 4·36 %), 4·09 % (95 % CI: 3·81 %, 4·36 %) and 3·97 % (95 % CI: 3·42 %, 4·53 %), respectively (online Supplementary Table S5).

**Fig. 5:**
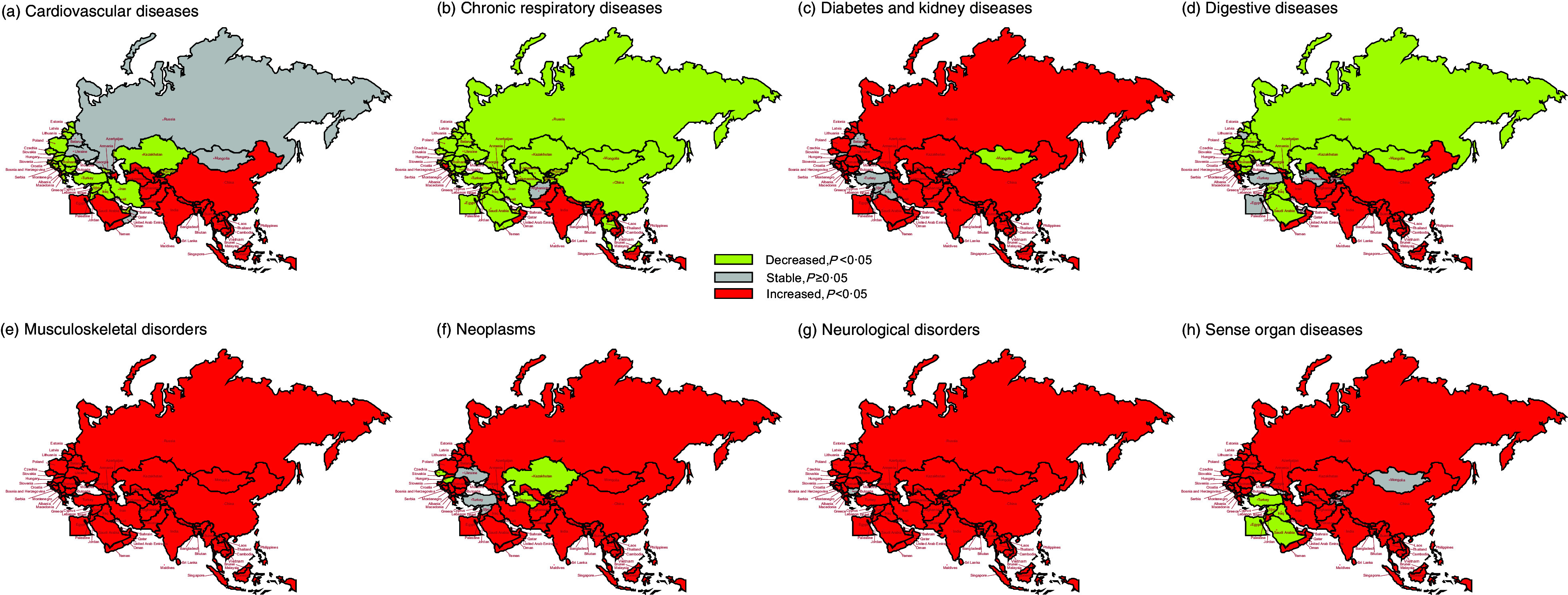
The temporal trend in the DALY rate of attributed to hBMI, stratified by disease for 1990–2019 in the BRI countries. (a) CVD. (b) Chronic respiratory diseases. (c) Diabetes and kidney diseases. (d) Digestive diseases. (e) Musculoskeletal disorders. (f) Neoplasms. (g) Neurological disorders. (h) Sensory organ diseases. DALY, disability-adjusted life years; hBMI, high BMI; BRI, Belt and Road Initiative

Figure 5 depicts the temporal trends of age-standardised DALY of hBMI attributed to various diseases. From 1990 to 2019, most BRI countries witnessed a significant increase in DALY for diabetes and kidney diseases, musculoskeletal disorders, neoplasms, neurological disorders and sensory organ diseases due to hBMI. Regarding DALY attributed to hBMI-related CVD, those countries experiencing increased DALY were predominantly in East Asia, South Asia and Southeast Asia, while those BRI countries showing a decline were mainly in Europe. In terms of DALY attributed to hBMI-related chronic respiratory diseases, countries such as India, Nepal, Maldives, Sri Lanka, Thailand, Oman, Bosnia and Herzegovina, and Montenegro exhibited an upward trend (all *P* < 0·05), while other BRI countries demonstrated a decrease or stability. For DALY attributed to hBMI-related diabetes and kidney diseases, only Mongolia and Cyprus displayed a downward trend (all *P* < 0·05) whereas other BRI countries showed an upward or stable pattern.

In the category of DALY attributed to hBMI-related digestive diseases, an upward trend was noted in regions such as East Asia (particularly in China), South Asia, Southeast Asia, high-income Asia Pacific and some European countries. Conversely, most countries showed a decline or remained stable in this aspect. In particular, Bahrain, Mongolia and Poland experienced significant decreases, with AAPC values of –2·62 % (95 % CI: –2·94 %, –2·30 %), –1·96 % (95 % CI: –2·18 %, –1·74 %) and –1·73 % (95 % CI: –1·97 %, –1·50 %), respectively (online Supplementary Table S6).

As for DALY attributed to hBMI-related neoplasms, only Kazakhstan, Kyrgyzstan, Bahrain, Czechia, Hungary and Israel demonstrated a decreasing trend (all *P* < 0·05), while other BRI countries showed an upward or stable pattern. In the case of DALY attributed to hBMI-related sensory organ diseases, the majority of countries in North Africa and the Middle East displayed a downward trend, while other BRI countries exhibited an upward or stable trend.

## Discussion

In this study, we aimed to investigate the trends and patterns of DB-hBMI across BRI member countries from 1990 to 2019. Our analysis provides valuable insights into the evolving landscape of hBMI-induced diseases in these countries.

Between 1990 and 2019, notable disparities in the trends of DALY due to hBMI were seen among countries in North Africa, the Middle East, Europe and Asia. By 1990, regions such as North Africa, the Middle East and Europe were already facing significant health burdens due to hBMI; this was largely attributable to early adoption of Western dietary patterns, sedentary lifestyles and urbanisation. However, in the following years, there was a marked decrease in DALY caused by hBMI, reflecting joint efforts towards enhancing public health awareness and implementing intervention measures^(20,21)^. This decline also highlights the value of Europe’s long-term investments in healthcare infrastructure, systems and public health initiatives^(22,23)^. Despite these positive developments, absolute numbers of DALY attributed to hBMI remain high in North Africa, the Middle East and Europe, underscoring the necessity to continue and intensify efforts to address the increasing prevalence of hBMI, especially in populous countries. In stark contrast to the downward trends observed in North Africa and the Middle East, South Asia and Southeast Asia have seen an upward trajectory in age-standardised DALY associated with hBMI. Countries like India, Nepal and Bangladesh have experienced significant increases in health burdens related to hBMI. These trends are likely influenced by a complex interplay of factors, including rapid urbanisation, shifts in dietary patterns and variations in healthcare accessibility^(24)^. Addressing the rising health burdens in these regions poses a formidable challenge.

In our analysis, we observed distinct trends in age-standardised DALY due to hBMI across different regions and genders. For males, there has been a notable increase in DALY across East Asia, South Asia, Southeast Asia and Central Asia, suggesting a rising health impact of hBMI. Southeast Asia and South Asia, particularly countries like Vietnam, Nepal and Bangladesh, has seen the sharpest increases, likely reflecting poor nutritional habits, low physical activity levels and unhealthy lifestyle choices prevalent in these regions^(25)^. In contrast, females in certain areas such as North Africa, the Middle East and parts of Europe, including Eastern and Central Europe, have experienced a decrease in age-standardised DALY related to hBMI. This gender difference might be attributed to factors like body fat distribution, with females generally having fat stored in less metabolically risky areas compared to males who generally store more abdominal or visceral fat than females^(26)^. Additionally, the level of physical activity among women in different regions is significantly influenced by cultural and social norms. In some underdeveloped areas of Asia and Africa, restrictions on women’s participation in sports and exercise may increase their susceptibility to health risks associated with hBMI^(27)^. In contrast, European regions actively encourage female participation in physical activities, which supports healthier lifestyle choices and contributes to lower BMI levels^(27)^. These observations underline the complex interplay of dietary habits, lifestyle choices and sociocultural factors in shaping the health outcomes related to hBMI^(28)^. Moreover, it highlights the necessity for gender-sensitive and region-specific public health strategies to effectively address the challenges posed by hBMI.

Between 1990 and 2019, China and South Asia experienced a significant rise in DALY due to hBMI, signalling a health crisis across various age groups. This surge is particularly alarming among youth aged 20–54 years; the increased DALY among youth underscore the escalating obesity epidemic and its health consequences. The primary drivers of this trend include sedentary lifestyles, unhealthy dietary habits and restricted healthcare access, further aggravated by the rapid pace of urbanisation^(29,30)^. The growing prevalence of obesity among the youth not only poses immediate health risks but also threatens long-term economic stability and healthcare system sustainability. Among older adults aged 50–74 years, the increased DALY reflect the compounded effects of chronic diseases associated with hBMI, such as diabetes and cardiovascular conditions^(31)^. Ageing increases vulnerability to these conditions, underscoring the critical need for effective preventive healthcare measures and robust healthcare infrastructure to mitigate this burden. For those aged 75 years and above, the rise in DALY is influenced by the general demographic trend towards an older population structure in developed and developing countries. Indeed, advances in healthcare and living standards have led to longer life expectancies, thereby increasing the proportion of elderly individuals in populations^(32)^. This shift emphasises the ageing society as a key factor in the growing DALY related to hBMI, as older adults are more prone to the adverse health impacts of hBMI due to age-related metabolic changes^(32)^. The observed increase in DALY across all age groups in China and South Asia highlights the urgent need for adaptable healthcare strategies tailored to the region’s evolving demographic profile.

The significant increase in age-standardised DALY attributed to hBMI from 1990 to 2019 across various disease categories, including diabetes and kidney diseases, musculoskeletal disorders, cancer, neurological disorders and sensory organ diseases, in most BRI countries, reflects the comprehensive impact of hBMI on health. First, hBMI is closely linked to diabetes and kidney diseases^(33)^. It leads to insulin resistance and impaired pancreatic function, increasing the risk of diabetes^(4)^. Diabetes, in turn, can damage kidney function, so hBMI indirectly contributes to kidney diseases. These two conditions interact, making it easier for individuals with hBMI to develop kidney-related complications^(34)^. Second, hBMI negatively affects the musculoskeletal system. Excessive weight or obesity places greater stress on joints, leading to joint pain, fractures and other musculoskeletal issues^(35)^. These problems can limit mobility, further exacerbating the health burden in hBMI individuals. Third, hBMI significantly raises the risk of cancer. Obesity is associated with various cancers, including breast cancer, endometrial cancer and colorectal cancer. This association may be due to the presence of adipose tissue, which can trigger chronic inflammation and hormonal imbalances, promoting the growth of cancer cells^(5)^. Finally, hBMI has adverse effects on the neurological system and sensory organs. Obesity is linked to neurological disorders like Parkinson’s disease and sensory issues such as vision impairment and hearing loss. Factors such as inflammation, metabolic disruptions and lipid deposits may contribute to the development of these neurological and sensory problems^(36)^. hBMI not only impacts one system or organ; it triggers chronic diseases through multiple pathways^(33)^. These include chronic inflammation, metabolic disturbances, hormonal imbalances, among others, which interact to promote the development of various diseases^(33)^. Consequently, developing comprehensive public health policies and individual health management plans to address the multifaceted impact of hBMI on health is essential.

The BRI encompasses a vast and geographically diverse part of the world, and, as evidenced by the study results, there are notable disparities among BRI countries in terms of the impact of hBMI on various diseases. These regional variations stem from a complex interplay of socio-economic, cultural and healthcare infrastructure factors. Addressing the public health challenges posed by hBMI in BRI countries necessitates robust international collaboration^(2)^. Such collaboration should extend beyond borders and encompass a range of strategies. First, countries within the BRI should establish formal mechanisms for sharing best practices and lessons learned in managing and preventing hBMI-related diseases. This could involve regular conferences, workshops and knowledge-sharing platforms where experts and policymakers come together to exchange insights. Second, collaborative epidemiological studies can provide a more comprehensive understanding of the factors contributing to hBMI and its health impacts across the BRI countries. These studies can help identify common trends and shared risk factors, which can inform the development of region-specific preventive measures. Furthermore, pooling resources and expertise for healthcare infrastructure development can address healthcare accessibility issues, particularly in less developed regions of the BRI. International funding and support can play a pivotal role in upgrading healthcare facilities and training healthcare professionals.

This study also has several limitations. First, the analysis relies primarily on data from the GBD study, which compiles data from various sources, including surveys, censuses and hospital records. While the GBD study is a valuable resource, it may still be subject to data inaccuracies, misreporting or underreporting in some regions, potentially affecting the accuracy of our findings. Second, the study focuses on BRI countries that have a wide range of economic, cultural and geographical differences. While we have attempted to provide a comprehensive analysis, there may be nuances within individual countries or subregions that our study may not have fully captured. Sub-national variations in disease prevalence, healthcare infrastructure and public health policies could impact the overall trends observed. Nonetheless, we believe that this study provides valuable insights into the relationship between hBMI and disease burden, offering valuable information for future public health policies and interventions.

### Conclusions

Our study spanning BRI countries from 1990 to 2019 reveals a concerning trend in DB-hBMI. While some countries have experienced declining trends in DALY from hBMI, others, particularly in South Asia and Southeast Asia, have seen an alarming rise. This calls for targeted interventions to address the multifaceted impact of hBMI on health. Tailoring healthcare strategies to the unique challenges faced by each region is essential, considering sociocultural and economic factors that contribute to these disparities. Therefore, collaboration among BRI countries is essential to devise and enact effective measures aimed at alleviating the impact of hBMI-induced diseases.

## Supporting information

Xu et al. supplementary material 1 Xu et al. supplementary material

Xu et al. supplementary material 2 Xu et al. supplementary material

Xu et al. supplementary material 3 Xu et al. supplementary material

Xu et al. supplementary material 4 Xu et al. supplementary material

Xu et al. supplementary material 5 Xu et al. supplementary material

Xu et al. supplementary material 6 Xu et al. supplementary material

## Supplementary material

For supplementary material accompanying this paper visit https://doi.org/10.1017/S1368980024001253

## References

[1] Tambo E , Khayeka-Wandabwa C , Muchiri GW et al. (2019) China’s Belt and Road Initiative: incorporating public health measures toward global economic growth and shared prosperity. Glob Health J 3, 46–49. 32501414 10.1016/j.glohj.2019.06.003 PMC7148655

[2] Tang K , Li Z , Li W et al. (2017) China’s silk road and global health. Lancet 390, 2595–2601. 29231838 10.1016/S0140-6736(17)32898-2 PMC7159269

[3] The Lancet Gastroenterology H (2021) Obesity: another ongoing pandemic. Lancet Gastroenterol Hepatol 6, 411. 34015350 10.1016/S2468-1253(21)00143-6 PMC9259282

[4] Piché ME , Tchernof A & Després JP (2020) Obesity phenotypes, diabetes, and cardiovascular diseases. Circ Res 126, 1477–1500. 32437302 10.1161/CIRCRESAHA.120.316101

[5] Avgerinos KI , Spyrou N , Mantzoros CS et al. (2019) Obesity and cancer risk: emerging biological mechanisms and perspectives. Metabolism 92, 121–135. 30445141 10.1016/j.metabol.2018.11.001

[6] Wearing SC , Hennig EM , Byrne NM et al. (2006) Musculoskeletal disorders associated with obesity: a biomechanical perspective. Obes Rev 7, 239–250. 16866972 10.1111/j.1467-789X.2006.00251.x

[7] Blüher M (2019) Obesity: global epidemiology and pathogenesis. Nat Rev Endocrinol 15, 288–298. 30814686 10.1038/s41574-019-0176-8

[8] Abdelaal M , le Roux CW & Docherty NG (2017) Morbidity and mortality associated with obesity. Ann Transl Med 5, 161. 10.21037/atm.2017.03.107 PMC5401682 28480197

[9] Kivimäki M , Strandberg T , Pentti J et al. (2022) Body-mass index and risk of obesity-related complex multimorbidity: an observational multicohort study. Lancet Diabetes Endocrinol 10, 253–263. 35248171 10.1016/S2213-8587(22)00033-X PMC8938400

[10] Jia P & Wang Y (2019) Global health efforts and opportunities related to the Belt and Road Initiative. Lancet Glob Health 7, e703–e705. 31097271 10.1016/S2214-109X(19)30062-2

[11] Popkin BM , Corvalan C & Grummer-Strawn LM (2020) Dynamics of the double burden of malnutrition and the changing nutrition reality. Lancet 395, 65–74. 31852602 10.1016/S0140-6736(19)32497-3 PMC7179702

[12] GBD 2019 Diseases and Injuries Collaborators (2020) Global burden of 369 diseases and injuries in 204 countries and territories, 1990–2019: a systematic analysis for the Global Burden of Disease Study 2019. Lancet 396, 1204–1222. 33069326 10.1016/S0140-6736(20)30925-9 PMC7567026

[13] GBD 2019 Meningitis Antimicrobial Resistance Collaborators (2023) Global, regional, and national burden of meningitis and its aetiologies, 1990–2019: a systematic analysis for the Global Burden of Disease Study 2019. Lancet Neurol 22, 685–711. 37479374 10.1016/S1474-4422(23)00195-3 PMC10356620

[14] Zhang Y , Luo Z , Yi J et al. (2023) Burden and trends of stroke attributable to dietary risk factors from 1990 to 2019 in the Belt and Road Initiative countries: an analysis from the global burden of disease study 2019. Front Nutr 10, 1235271. 37565042 10.3389/fnut.2023.1235271 PMC10410448

[15] Andersen LB (2007) Physical activity and health. BMJ (Clin Res Ed) 334, 1173. 10.1136/bmj.39225.414537.80 PMC1889998 17556432

[16] National Bureau of Statistics (2023) How does the World Bank Divide Economies’ Income Levels. http://www.stats.gov.cn/zsk/snapshoot?reference=33e2b9cdb6391521c53328be6244e40b_E6DA54C3D7C349E7DA6040449FF84CE7&siteCode=tjzsk (accessed November 2023).

[17] Roth GA , Mensah GA , Johnson CO et al. (2020) Global burden of cardiovascular diseases and risk factors, 1990–2019: update from the GBD 2019 study. J Am Coll Cardiol 76, 2982–3021. 33309175 10.1016/j.jacc.2020.11.010 PMC7755038

[18] Clegg LX , Hankey BF , Tiwari R et al. (2009) Estimating average annual per cent change in trend analysis. Stat Med 28, 3670–3682. 19856324 10.1002/sim.3733 PMC2843083

[19] GBD 2019 Stroke Collaborators (2021) Global, regional, and national burden of stroke and its risk factors, 1990–2019: a systematic analysis for the Global Burden of Disease Study 2019. Lancet Neurol 20, 795–820. 34487721 10.1016/S1474-4422(21)00252-0 PMC8443449

[20] Shibli H , Aharonson-Daniel L & Feder-Bubis P (2021) Perceptions about the accessibility of healthcare services among ethnic minority women: a qualitative study among Arab Bedouins in Israel. Int J Equity Health 20, 117. 33964946 10.1186/s12939-021-01464-9 PMC8106134

[21] Mannucci PM & Peyvandi F (2023) Air pollution and cardiovascular health in Middle East and North Africa: many shadows but some light. Eur J Prev Cardiol 30, 254–255. 36515623 10.1093/eurjpc/zwac292

[22] Stenzinger A , Moltzen EK , Winkler E et al. (2023) Implementation of precision medicine in healthcare-a European perspective. J Intern Med 294, 437–454. 37455247 10.1111/joim.13698

[23] Greer SL , Hervey TK , Mackenbach JP et al. (2013) Health law and policy in the European Union. Lancet 381, 1135–1144. 23541054 10.1016/S0140-6736(12)62083-2

[24] Ng M , Fleming T , Robinson M et al. (2014) Global, regional, and national prevalence of overweight and obesity in children and adults during 1980–2013: a systematic analysis for the Global Burden of Disease Study 2013. Lancet 384, 766–781. 24880830 10.1016/S0140-6736(14)60460-8 PMC4624264

[25] Khandelwal S & Reddy KS (2013) Eliciting a policy response for the rising epidemic of overweight-obesity in India. Obes Rev 14, 114–125. 24103051 10.1111/obr.12097

[26] Karastergiou K , Smith SR , Greenberg AS et al. (2012) Sex differences in human adipose tissues – the biology of pear shape. Biol Sex Differ 3, 13. 22651247 10.1186/2042-6410-3-13 PMC3411490

[27] Coleman L , Cox L & Roker D (2008) Girls and young women’s participation in physical activity: psychological and social influences. Health Educ Res 23, 633–647. 17897930 10.1093/her/cym040

[28] Zaragoza J , Generelo E , Julián JA et al. (2011) Barriers to adolescent girls’ participation in physical activity defined by physical activity levels. J Sports Med Phys Fitness 51, 128–135. 21297572

[29] Gore FM , Bloem PJ , Patton GC et al. (2011) Global burden of disease in young people aged 10–24 years: a systematic analysis. Lancet 377, 2093–2102. 21652063 10.1016/S0140-6736(11)60512-6

[30] Powell-Wiley TM , Poirier P , Burke LE et al. (2021) Obesity and cardiovascular disease: a scientific statement from the American Heart Association. Circulation 143, e984–e1010. 33882682 10.1161/CIR.0000000000000973 PMC8493650

[31] Palmer AK & Jensen MD (2022) Metabolic changes in aging humans: current evidence and therapeutic strategies. J Clin Invest 132, e158451. 35968789 10.1172/JCI158451 PMC9374375

[32] Bloom DE & Luca DL (2016) Chapter 1 - The global demography of aging: facts, explanations, future. In Handbook of the Economics of Population Aging, vol. 1, pp. 3–56 [ J Piggott and A Woodland , editors]. North-Holland: Elsevier.

[33] Larsson SC & Burgess S (2021) Causal role of high body mass index in multiple chronic diseases: a systematic review and meta-analysis of Mendelian randomization studies. BMC Med 19, 320. 34906131 10.1186/s12916-021-02188-x PMC8672504

[34] Kjaergaard AD , Teumer A , Witte DR et al. (2022) Obesity and kidney function: a two-sample Mendelian randomization study. Clin Chem 68, 461–472. 34922334 10.1093/clinchem/hvab249 PMC7614591

[35] Anandacoomarasamy A , Caterson I , Sambrook P et al. (2008) The impact of obesity on the musculoskeletal system. Int J Obes (Lond) 32, 211–222. 17848940 10.1038/sj.ijo.0803715

[36] O’Brien PD , Hinder LM , Callaghan BC et al. (2017) Neurological consequences of obesity. Lancet Neurol 16, 465–477. 28504110 10.1016/S1474-4422(17)30084-4 PMC5657398

