# Supplementary material for: Spatial and temporal patterns of disease burden attributable to high BMI in Belt and Road Initiative countries, 1990–2019
Source: Public Health Nutr. 2024 Jun 5;27(1):e158. doi: 10.1017/S1368980024001253 (PMC11617424; doi:10.1017/S1368980024001253)
Supplement: Xu et al. supplementary material 2 — Xu et al. supplementary material [file S1368980024001253sup002.docx]

**Table S2** The age-standardized mortality, YLDs, YLLs and DALYs attributed to high BMI for the BRI countries in 1990 and 2019

|  |  | **1990(/100,000)** |  |  |  |  | **2019(/100,000)** |  |  |
| --- | --- | --- | --- | --- | --- | --- | --- | --- | --- |
| **Gobal/Regions/Countries** | **Mortality** | **YLDs** | **YLLs** | **DALYs** |  | **Mortality** | **YLDs** | **YLLs** | **DALYs** |
| Global | 59.65 | 307.46 | 1330.13 | 1637.58 |  | 62.59 | 492.42 | 1440.12 | 1932.54 |
| **SDI Regions** |  |  |  |  |  |  |  |  |  |
| High SDI | 60.56 | 460.38 | 1301.86 | 1762.23 |  | 45.65 | 652.44 | 978.66 | 1631.11 |
| High-middle SDI | 82.01 | 378.76 | 1796.72 | 2175.49 |  | 69.14 | 514.95 | 1466.88 | 1981.83 |
| Middle SDI | 49.34 | 236.39 | 1192.88 | 1429.27 |  | 68.92 | 486.48 | 1632.10 | 2118.58 |
| Low-middle SDI | 32.58 | 141.24 | 807.22 | 948.46 |  | 60.34 | 384.89 | 1507.31 | 1892.20 |
| Low SDI | 37.44 | 134.40 | 961.20 | 1095.60 |  | 55.55 | 294.92 | 1403.22 | 1698.14 |
| **World Bank Regions** |  |  |  |  |  |  |  |  |  |
| World Bank High Income | 63.72 | 467.18 | 1371.04 | 1838.22 |  | 47.24 | 652.75 | 992.14 | 1644.89 |
| World Bank Upper Middle Income | 62.95 | 288.36 | 1429.57 | 1717.94 |  | 63.35 | 477.44 | 1390.44 | 1867.88 |
| World Bank Lower Middle Income | 47.07 | 184.84 | 1106.04 | 1290.89 |  | 72.46 | 426.98 | 1793.95 | 2220.93 |
| World Bank Low Income | 44.33 | 149.77 | 1140.38 | 1290.15 |  | 60.13 | 293.57 | 1495.54 | 1789.11 |
| **East Asia** |  |  |  |  |  |  |  |  |  |
| China | 29.80 | 142.86 | 709.44 | 852.30 |  | 40.55 | 321.50 | 909.44 | 1230.94 |
| **Central Asia** |  |  |  |  |  |  |  |  |  |
| Armenia | 86.17 | 388.01 | 1908.44 | 2296.45 |  | 111.40 | 693.24 | 2328.46 | 3021.70 |
| Azerbaijan | 114.62 | 404.25 | 2786.80 | 3191.06 |  | 182.94 | 693.74 | 3769.34 | 4463.08 |
| Georgia | 164.88 | 488.73 | 3805.02 | 4293.75 |  | 139.08 | 723.33 | 3074.55 | 3797.89 |
| Kazakhstan | 138.84 | 559.30 | 3168.79 | 3728.10 |  | 147.27 | 809.46 | 3130.45 | 3939.91 |
| Kyrgyzstan | 95.52 | 381.75 | 2409.89 | 2791.64 |  | 104.73 | 466.33 | 2356.51 | 2822.84 |
| Mongolia | 142.49 | 322.84 | 3638.80 | 3961.65 |  | 156.57 | 426.55 | 3922.35 | 4348.90 |
| Tajikistan | 58.20 | 206.99 | 1443.75 | 1650.74 |  | 106.16 | 391.42 | 2345.50 | 2736.92 |
| Turkmenistan | 137.70 | 429.47 | 3352.96 | 3782.43 |  | 182.09 | 718.47 | 4418.01 | 5136.48 |
| Uzbekistan | 101.62 | 382.19 | 2495.20 | 2877.39 |  | 228.74 | 672.20 | 4920.92 | 5593.13 |
| **South Asia** |  |  |  |  |  |  |  |  |  |
| Bangladesh | 13.42 | 53.31 | 365.50 | 418.81 |  | 33.29 | 233.99 | 915.50 | 1149.49 |
| Bhutan | 29.41 | 136.05 | 774.99 | 911.04 |  | 55.74 | 385.61 | 1347.98 | 1733.59 |
| India | 24.67 | 117.30 | 627.20 | 744.49 |  | 51.60 | 383.17 | 1314.67 | 1697.84 |
| Nepal | 15.25 | 66.35 | 401.12 | 467.47 |  | 42.15 | 310.15 | 1044.18 | 1354.34 |
| Pakistan | 34.88 | 156.01 | 874.99 | 1030.99 |  | 92.25 | 447.24 | 2330.40 | 2777.63 |
| **Southeast Asia** |  |  |  |  |  |  |  |  |  |
| Cambodia | 23.18 | 88.01 | 644.03 | 732.05 |  | 44.35 | 288.50 | 1124.39 | 1412.89 |
| Indonesia | 30.09 | 145.18 | 872.07 | 1017.24 |  | 80.48 | 455.61 | 2244.25 | 2699.86 |
| Lao | 35.78 | 129.25 | 980.38 | 1109.63 |  | 77.37 | 445.62 | 2075.83 | 2521.45 |
| Malaysia | 59.28 | 349.32 | 1579.66 | 1928.97 |  | 72.34 | 655.18 | 1778.96 | 2434.14 |
| Maldives | 32.80 | 125.58 | 892.21 | 1017.79 |  | 35.54 | 376.09 | 852.97 | 1229.06 |
| Burma | 34.19 | 101.85 | 989.16 | 1091.02 |  | 65.95 | 381.27 | 1771.11 | 2152.38 |
| Philippines | 33.29 | 220.44 | 814.90 | 1035.34 |  | 82.66 | 428.48 | 2174.01 | 2602.50 |
| Sri Lanka | 42.02 | 242.39 | 1002.34 | 1244.73 |  | 66.84 | 659.89 | 1413.50 | 2073.38 |
| Thailand | 27.27 | 186.71 | 734.48 | 921.19 |  | 43.34 | 474.76 | 1132.86 | 1607.62 |
| Viet Nam | 16.24 | 67.71 | 393.10 | 460.80 |  | 42.49 | 258.18 | 990.27 | 1248.45 |
| **High-income Asia pacific** |  |  |  |  |  |  |  |  |  |
| Brunei | 64.21 | 340.32 | 1447.19 | 1787.51 |  | 72.05 | 649.05 | 1538.51 | 2187.56 |
| Singapore | 32.88 | 270.76 | 784.32 | 1055.09 |  | 23.11 | 449.73 | 496.20 | 945.93 |
| **North Africa and Middle East** |  |  |  |  |  |  |  |  |  |
| Afghanistan | 132.74 | 315.85 | 3607.87 | 3923.72 |  | 177.28 | 670.19 | 4428.42 | 5098.61 |
| Bahrain | 211.59 | 807.07 | 4646.44 | 5453.51 |  | 161.71 | 1318.99 | 2978.40 | 4297.40 |
| Egypt | 173.76 | 454.87 | 4193.36 | 4648.23 |  | 217.70 | 852.49 | 5077.10 | 5929.58 |
| Iran | 89.20 | 392.74 | 2026.84 | 2419.58 |  | 91.72 | 716.76 | 1864.16 | 2580.92 |
| Iraq | 194.46 | 743.58 | 4769.06 | 5512.65 |  | 172.01 | 942.31 | 3850.80 | 4793.11 |
| Jordan | 178.39 | 683.99 | 3911.68 | 4595.67 |  | 137.12 | 976.64 | 2725.00 | 3701.63 |
| Kuwait | 122.15 | 790.97 | 2790.51 | 3581.48 |  | 93.59 | 1167.17 | 1988.91 | 3156.08 |
| Lebanon | 128.15 | 550.48 | 2948.88 | 3499.35 |  | 120.60 | 923.79 | 2564.81 | 3488.60 |
| Oman | 114.05 | 369.81 | 2807.66 | 3177.47 |  | 177.53 | 928.86 | 3472.82 | 4401.68 |
| Palestine | 122.16 | 476.38 | 2942.73 | 3419.11 |  | 131.57 | 789.84 | 2857.53 | 3647.36 |
| Qatar | 229.42 | 904.03 | 4610.90 | 5514.94 |  | 209.62 | 1587.82 | 3317.12 | 4904.93 |
| Saudi Arabia | 121.56 | 544.88 | 2941.14 | 3486.02 |  | 160.71 | 1063.64 | 3707.90 | 4771.54 |
| Syrian Arab Republic | 141.86 | 506.41 | 3595.68 | 4102.09 |  | 143.80 | 753.58 | 3240.93 | 3994.50 |
| Turkey | 127.59 | 561.25 | 2909.00 | 3470.25 |  | 95.18 | 783.45 | 1879.17 | 2662.62 |
| United Arab Emirates | 224.55 | 841.07 | 5036.73 | 5877.80 |  | 203.03 | 1317.70 | 4415.22 | 5732.92 |
| Yemen | 64.31 | 176.59 | 1663.27 | 1839.85 |  | 88.64 | 379.94 | 2215.70 | 2595.64 |
| **Central Europe** |  |  |  |  |  |  |  |  |  |
| Albania | 74.60 | 354.64 | 1528.41 | 1883.05 |  | 74.11 | 535.45 | 1474.32 | 2009.78 |
| Bosnia and Herzegovina | 83.02 | 465.72 | 1842.00 | 2307.72 |  | 101.53 | 847.03 | 1988.79 | 2835.82 |
| Bulgaria | 175.15 | 644.86 | 3897.29 | 4542.15 |  | 159.50 | 763.43 | 3479.45 | 4242.88 |
| Croatia | 116.02 | 591.78 | 2449.03 | 3040.81 |  | 85.25 | 780.40 | 1570.28 | 2350.68 |
| Czechia | 135.06 | 666.44 | 2950.72 | 3617.16 |  | 80.20 | 998.21 | 1471.04 | 2469.25 |
| Hungary | 150.94 | 706.08 | 3473.88 | 4179.96 |  | 103.79 | 846.45 | 2090.11 | 2936.56 |
| Montenegro | 115.21 | 685.76 | 2589.04 | 3274.80 |  | 127.71 | 897.42 | 2615.42 | 3512.84 |
| Macedonia | 149.34 | 641.07 | 3186.66 | 3827.73 |  | 162.73 | 927.94 | 3119.86 | 4047.80 |
| Poland | 127.10 | 653.03 | 2873.52 | 3526.56 |  | 80.42 | 798.45 | 1597.78 | 2396.24 |
| Romania | 129.31 | 557.11 | 2784.62 | 3341.73 |  | 111.85 | 737.39 | 2295.55 | 3032.94 |
| Serbia | 142.05 | 632.71 | 3067.88 | 3700.59 |  | 126.38 | 862.94 | 2396.35 | 3259.29 |
| Slovakia | 136.35 | 584.94 | 3047.96 | 3632.90 |  | 96.14 | 726.55 | 1851.34 | 2577.89 |
| Slovenia | 85.91 | 589.52 | 1776.66 | 2366.18 |  | 54.03 | 695.36 | 950.50 | 1645.86 |
| **Eastern Europe** |  |  |  |  |  |  |  |  |  |
| Belarus | 100.85 | 461.04 | 2255.49 | 2716.53 |  | 121.19 | 562.44 | 2598.14 | 3160.58 |
| Estonia | 120.52 | 490.65 | 2652.06 | 3142.71 |  | 104.63 | 655.53 | 1885.04 | 2540.56 |
| Latvia | 124.10 | 534.46 | 2759.03 | 3293.49 |  | 104.88 | 683.58 | 2120.06 | 2803.64 |
| Lithuania | 104.05 | 479.78 | 2248.38 | 2728.17 |  | 95.99 | 585.85 | 1890.56 | 2476.40 |
| Moldova | 116.52 | 474.02 | 2550.22 | 3024.24 |  | 116.59 | 651.20 | 2472.78 | 3123.98 |
| Russian Federation | 119.49 | 476.99 | 2656.08 | 3133.06 |  | 122.61 | 593.32 | 2670.00 | 3263.32 |
| Ukraine | 123.16 | 534.08 | 2602.27 | 3136.36 |  | 155.58 | 603.32 | 3443.73 | 4047.05 |
| **Western Europe** |  |  |  |  |  |  |  |  |  |
| Cyprus | 71.03 | 339.76 | 1304.00 | 1643.76 |  | 46.46 | 473.50 | 789.02 | 1262.52 |
| Greece | 63.11 | 381.63 | 1270.81 | 1652.44 |  | 50.12 | 515.48 | 998.05 | 1513.53 |
| Israel | 68.48 | 385.52 | 1368.83 | 1754.35 |  | 41.68 | 511.06 | 733.56 | 1244.62 |

(YLDs, years lived with disability; YLLs, Years of Life Lost; DALYs, disability-adjusted life-years; BMI, Body Mass Index; BRI, Belt and Road Initiative.)
