# Supplementary material for: Spatial and temporal patterns of disease burden attributable to high BMI in Belt and Road Initiative countries, 1990–2019
Source: Public Health Nutr. 2024 Jun 5;27(1):e158. doi: 10.1017/S1368980024001253 (PMC11617424; doi:10.1017/S1368980024001253)
Supplement: Xu et al. supplementary material 3 — Xu et al. supplementary material [file S1368980024001253sup003.docx]

**Table S3** The average annual percentage change (AAPC) of mortality and DALY rates attributed to high BMI for 1990-2019 and 2010-2019 in the BRI countries

|  |  | **Mortality** |  |  |  |  |  | **DALYs** |  |  |  |
| --- | --- | --- | --- | --- | --- | --- | --- | --- | --- | --- | --- |
|  | **1990—2019** |  |  | **2010—2019** |  |  | **1990-2019** |  |  | **2010-2019** |  |
| **Countries** | **AAPC *95%CI*** | ***P* value** |  | **AAPC *95%CI*** | ***P* value** |  | **AAPC *95%CI*** | ***P* value** |  | **AAPC *95%CI*** | ***P* value** |
| Global | 0.03(-0.03,0.10) | 0.292 |  | 0.20(0.09,0.31) | 0.003 |  | 0.48(0.43,0.52) | <0.001 |  | 0.65(0.55,0.74) | <0.001 |
| **SDI levels** |  |  |  |  |  |  |  |  |  |  |  |
| High SDI | -1.18(-1.27,-1.10) | <0.001 |  | -0.31(-0.51,-0.11) | 0.007 |  | -0.33(-0.39,-0.28) | <0.001 |  | 0.23(0.08,0.38) | 0.008 |
| High middle SDI | -0.90(-1.10,-0.69) | <0.001 |  | -1.27(-1.57,-0.97) | <0.001 |  | -0.63(-0.82,-0.45) | <0.001 |  | -0.86(-1.15,-0.56) | <0.001 |
| Middle SDI | 1.21(1.16,1.27) | <0.001 |  | 0.82(0.70,0.93) | <0.001 |  | 1.41(1.36,1.46) | <0.001 |  | 1.12(1.04,1.20) | <0.001 |
| Low middle SDI | 2.26(2.20,2.32) | <0.001 |  | 2.02(1.80,2.25) | <0.001 |  | 2.53(2.48,2.58) | <0.001 |  | 2.29(2.07,2.51) | <0.001 |
| Low SDI | 1.46(1.40,1.53) | <0.001 |  | 1.76(1.66,1.86) | <0.001 |  | 1.60(1.52,1.68) | <0.001 |  | 2.04(1.93,2.14) | <0.001 |
| **World Bank Income levels** |  |  |  |  |  |  |  |  |  |  |  |
| World Bank High Income | -1.24(-1.32,-1.16) | <0.001 |  | -0.42(-0.63,-0.21) | 0.002 |  | -0.48(-0.53,-0.43) | <0.001 |  | 0.10(-0.09,0.29) | 0.263 |
| World Bank Upper Middle Income | -0.16(-0.29,-0.02) | <0.001 |  | -0.51(-0.68,-0.34) | <0.001 |  | 0.10(-0.02,0.23) | <0.001 |  | -0.09(-0.27,0.08) | 0.257 |
| World Bank Lower Middle Income | 1.46(1.37,1.54) | <0.001 |  | 1.29(1.09,1.49) | <0.001 |  | 1.89(1.82,1.95) | <0.001 |  | 1.67(1.46,1.88) | <0.001 |
| World Bank Low Income | 1.14(0.98,1.30) | 0.025 |  | 1.98(1.89,2.06) | <0.001 |  | 1.20(1.03,1.38) | 0.098 |  | 2.14(2.03,2.24) | <0.001 |
| **East Asia** |  |  |  |  |  |  |  |  |  |  |  |
| China | 1.22(1.13,1.30) | <0.001 |  | 0.64(0.48,0.79) | <0.001 |  | 1.41(1.34,1.49) | <0.001 |  | 0.94(0.83,1.05) | <0.001 |
| **Central Asia** |  |  |  |  |  |  |  |  |  |  |  |
| Armenia | 0.66(0.47,0.85) | <0.001 |  | -0.63(-0.95,-0.30) | 0.002 |  | 0.81(0.63,1.00) | <0.001 |  | -0.53(-0.87,-0.19) | 0.007 |
| Azerbaijan | 1.74(1.54,1.93) | <0.001 |  | 0.51(-0.08,1.11) | 0.081 |  | 1.16(0.98,1.34) | <0.001 |  | 0.16(-0.11,0.43) | 0.206 |
| Georgia | -0.56(-0.74,-0.37) | <0.001 |  | -0.59(-1.20,0.01) | 0.053 |  | -0.53(-0.71,-0.36) | <0.001 |  | -0.74(-1.57,0.10) | 0.075 |
| Kazakhstan | -0.57(-1.13,0.00) | 0.051 |  | -2.38(-2.72,-2.04) | <0.001 |  | -0.58(-1.14,-0.01) | 0.047 |  | -2.23(-2.60,-1.86) | <0.001 |
| Kyrgyzstan | -0.01(-0.39,0.37) | 0.948 |  | -1.58(-1.85,-1.32) | <0.001 |  | -0.42(-0.77,-0.06) | 0.023 |  | -1.74(-2.09,-1.39) | <0.001 |
| Mongolia | -0.09(-0.44,0.25) | 0.580 |  | -0.99(-1.56,-0.41) | 0.004 |  | -0.09(-0.43,0.25) | 0.596 |  | -1.09(-1.63,-0.55) | 0.002 |
| Tajikistan | 2.06(1.73,2.39) | <0.001 |  | 3.06(2.07,4.05) | <0.001 |  | 1.64(1.25,2.04) | <0.001 |  | 3.01(2.26,3.76) | <0.001 |
| Turkmenistan | 0.52(0.20,0.83) | 0.002 |  | 1.18(0.78,1.58) | <0.001 |  | 0.70(0.40,1.00) | <0.001 |  | 1.11(0.65,1.57) | 0.001 |
| Uzbekistan | 2.71(2.21,3.21) | <0.001 |  | -0.09(-0.69,0.52) | 0.749 |  | 2.12(1.70,2.54) | <0.001 |  | 0.02(-0.38,0.43) | 0.899 |
| **South Asia** |  |  |  |  |  |  |  |  |  |  |  |
| Bangladesh | 3.94(3.51,4.37) | <0.001 |  | 1.20(0.46,1.95) | 0.006 |  | 4.42(4.00,4.83) | <0.001 |  | 1.36(1.01,1.72) | <0.001 |
| Bhutan | 2.29(2.16,2.43) | <0.001 |  | 1.77(1.70,1.85) | <0.001 |  | 2.28(2.13,2.44) | <0.001 |  | 1.63(1.60,1.67) | <0.001 |
| India | 2.56(2.38,2.74) | <0.001 |  | 2.63(2.30,2.97) | <0.001 |  | 2.90(2.79,3.01) | <0.001 |  | 2.82(2.48,3.16) | <0.001 |
| Nepal | 4.03(3.81,4.25) | <0.001 |  | 3.82(3.28,4.37) | <0.001 |  | 4.19(3.97,4.41) | <0.001 |  | 3.99(3.46,4.52) | <0.001 |
| Pakistan | 3.63(3.22,4.04) | <0.001 |  | 0.89(0.81,0.97) | <0.001 |  | 3.71(3.32,4.10) | <0.001 |  | 1.01(0.89,1.13) | <0.001 |
| **Southeast Asia** |  |  |  |  |  |  |  |  |  |  |  |
| Cambodia | 2.46(2.27,2.65) | <0.001 |  | 2.61(2.43,2.79) | <0.001 |  | 2.46(2.27,2.66) | <0.001 |  | 2.79(2.67,2.92) | <0.001 |
| Indonesia | 3.88(3.72,4.04) | <0.001 |  | 2.88(2.50,3.27) | <0.001 |  | 3.84(3.70,3.98) | <0.001 |  | 2.71(2.28,3.14) | <0.001 |
| Lao | 2.92(2.79,3.05) | <0.001 |  | 2.08(2.01,2.14) | <0.001 |  | 3.17(3.01,3.32) | <0.001 |  | 2.07(1.99,2.14) | <0.001 |
| Malaysia | 0.41(0.19,0.63) | 0.001 |  | 1.11(0.38,1.84) | 0.008 |  | 0.70(0.55,0.85) | <0.001 |  | 1.15(0.79,1.51) | <0.001 |
| Maldives | -0.18(-0.43,0.08) | 0.162 |  | 1.63(1.40,1.85) | <0.001 |  | 0.34(0.11,0.56) | 0.005 |  | 1.75(1.56,1.94) | <0.001 |
| Burma | 2.67(2.50,2.84) | <0.001 |  | 2.32(2.12,2.52) | <0.001 |  | 2.76(2.59,2.93) | <0.001 |  | 2.14(1.99,2.29) | <0.001 |
| Philippines | 3.54(3.21,3.88) | <0.001 |  | 2.52(1.68,3.38) | <0.001 |  | 3.52(3.18,3.87) | <0.001 |  | 2.48(1.75,3.22) | <0.001 |
| Sri Lanka | 1.94(1.74,2.15) | <0.001 |  | 0.30(0.00,0.59) | 0.047 |  | 1.99(1.83,2.14) | <0.001 |  | 0.90(0.76,1.04) | <0.001 |
| Thailand | 1.09(0.83,1.35) | <0.001 |  | 1.48(1.10,1.87) | <0.001 |  | 1.32(1.03,1.62) | <0.001 |  | 1.97(1.57,2.37) | <0.001 |
| Viet Nam | 4.14(3.81,4.48) | <0.001 |  | 2.80(2.36,3.23) | <0.001 |  | 4.28(3.92,4.64) | <0.001 |  | 3.08(2.66,3.49) | <0.001 |
| **High-income Asia pacific** |  |  |  |  |  |  |  |  |  |  |  |
| Brunei | 0.84(0.62,1.07) | <0.001 |  | -0.26(-0.45,-0.08) | 0.011 |  | 1.10(0.91,1.29) | <0.001 |  | 0.06(-0.05,0.17) | 0.270 |
| Singapore | -1.45(-1.61,-1.28) | <0.001 |  | -1.64(-1.92,-1.36) | <0.001 |  | -0.59(-0.74,-0.44) | <0.001 |  | -1.11(-1.39,-0.83) | <0.001 |
| **North Africa and Middle East** |  |  |  |  |  |  |  |  |  |  |  |
| Afghanistan | 1.47(0.98,1.96) | <0.001 |  | 2.30(1.91,2.70) | <0.001 |  | 1.34(0.88,1.81) | <0.001 |  | 2.11(1.72,2.50) | <0.001 |
| Bahrain | -0.96(-1.22,-0.69) | <0.001 |  | -1.82(-2.48,-1.17) | <0.001 |  | -1.01(-1.17,-0.85) | <0.001 |  | -1.05(-1.51,-0.58) | 0.001 |
| Egypt | 0.86(0.77,0.95) | <0.001 |  | 0.68(0.22,1.15) | 0.010 |  | 0.95(0.88,1.02) | <0.001 |  | 0.73(0.38,1.08) | 0.001 |
| Iran | -0.08(-0.23,0.06) | 0.245 |  | 0.82(0.54,1.11) | <0.001 |  | 0.07(-0.05,0.19) | 0.260 |  | 0.84(0.63,1.04) | <0.001 |
| Iraq | -0.59(-0.66,-0.51) | <0.001 |  | -0.27(-0.68,0.14) | 0.168 |  | -0.70(-0.76,-0.63) | <0.001 |  | -0.51(-0.83,-0.18) | 0.007 |
| Jordan | -1.27(-1.49,-1.04) | <0.001 |  | -0.67(-1.14,-0.20) | 0.011 |  | -1.10(-1.31,-0.89) | <0.001 |  | -0.51(-0.92,-0.10) | 0.021 |
| Kuwait | -0.62(-1.06,-0.19) | 0.007 |  | -0.71(-1.70,0.29) | 0.139 |  | -0.23(-0.60,0.14) | 0.220 |  | -0.40(-1.14,0.34) | 0.244 |
| Lebanon | 0.02(-0.21,0.26) | 0.836 |  | 0.29(-0.04,0.63) | 0.077 |  | 0.22(-0.04,0.48) | 0.097 |  | 0.70(0.46,0.94) | <0.001 |
| Oman | 1.69(1.30,2.09) | <0.001 |  | -1.24(-1.95,-0.51) | 0.004 |  | 1.17(0.71,1.62) | <0.001 |  | -1.94(-2.33,-1.56) | <0.001 |
| Palestine | -0.10(-0.32,0.12) | 0.345 |  | 1.92(1.21,2.63) | <0.001 |  | -0.13(-0.31,0.05) | 0.142 |  | 1.49(1.01,1.97) | <0.001 |
| Qatar | -0.17(-0.49,0.15) | 0.284 |  | -1.65(-2.08,-1.22) | <0.001 |  | -0.39(-0.70,-0.09) | 0.014 |  | -2.12(-2.34,-1.90) | <0.001 |
| Saudi Arabia | 0.82(0.51,1.13) | <0.001 |  | -1.10(-1.21,-0.99) | <0.001 |  | 1.00(0.74,1.27) | <0.001 |  | -0.73(-0.89,-0.58) | <0.001 |
| Syrian Arab Republic | -0.43(-0.66,-0.19) | 0.001 |  | 0.52(0.39,0.64) | <0.001 |  | -0.49(-0.70,-0.28) | <0.001 |  | 0.73(0.56,0.90) | <0.001 |
| Turkey | -1.10(-1.36,-0.84) | <0.001 |  | -0.43(-0.57,-0.29) | <0.001 |  | -0.99(-1.22,-0.77) | <0.001 |  | -0.29(-0.44,-0.14) | 0.002 |
| United Arab Emirates | -0.29(-0.82,0.25) | 0.287 |  | -4.14(-5.50,-2.76) | <0.001 |  | -0.06(-0.41,0.30) | 0.737 |  | -2.70(-3.58,-1.80) | <0.001 |
| Yemen | 1.46(1.30,1.62) | <0.001 |  | 1.23(0.79,1.68) | <0.001 |  | 1.51(1.37,1.65) | <0.001 |  | 1.31(0.91,1.72) | <0.001 |
| **Central Europe** |  |  |  |  |  |  |  |  |  |  |  |
| Albania | 0.25(0.04,0.46) | 0.020 |  | 0.86(0.65,1.08) | <0.001 |  | 0.49(0.30,0.68) | <0.001 |  | 1.06(0.91,1.21) | <0.001 |
| Bosnia and Herzegovina | 0.78(0.55,1.00) | <0.001 |  | 0.01(-0.26,0.27) | 0.961 |  | 0.75(0.59,0.91) | <0.001 |  | 0.09(-0.11,0.30) | 0.318 |
| Bulgaria | -0.93(-1.18,-0.69) | <0.001 |  | -0.06(-0.57,0.45) | 0.803 |  | -0.82(-1.05,-0.59) | <0.001 |  | 0.11(-0.35,0.56) | 0.606 |
| Croatia | -0.91(-1.00,-0.83) | <0.001 |  | -1.27(-1.71,-0.82) | <0.001 |  | -0.90(-0.97,-0.83) | <0.001 |  | -0.93(-1.33,-0.53) | 0.001 |
| Czechia | -1.88(-1.97,-1.79) | <0.001 |  | -1.04(-1.31,-0.77) | <0.001 |  | -1.42(-1.54,-1.29) | <0.001 |  | -0.38(-0.63,-0.14) | 0.007 |
| Hungary | -1.46(-1.56,-1.36) | <0.001 |  | -0.92(-1.32,-0.52) | 0.001 |  | -1.45(-1.55,-1.35) | <0.001 |  | -0.90(-1.29,-0.50) | 0.001 |
| Montenegro | 0.48(0.33,0.63) | <0.001 |  | -0.08(-0.46,0.30) | 0.638 |  | 0.25(0.09,0.42) | 0.003 |  | -0.13(-0.30,0.04) | 0.111 |
| Macedonia | 0.25(0.05,0.45) | 0.016 |  | -0.60(-0.79,-0.40) | <0.001 |  | 0.04(-0.13,0.21) | 0.623 |  | -0.40(-0.61,-0.20) | 0.002 |
| Poland | -1.89(-2.00,-1.78) | <0.001 |  | -0.99(-1.34,-0.63) | <0.001 |  | -1.58(-1.71,-1.45) | <0.001 |  | -0.71(-1.04,-0.38) | 0.001 |
| Romania | -0.95(-1.18,-0.71) | <0.001 |  | -1.21(-1.80,-0.61) | 0.002 |  | -0.81(-1.04,-0.57) | <0.001 |  | -0.88(-1.47,-0.29) | 0.009 |
| Serbia | -0.68(-0.88,-0.48) | <0.001 |  | -0.66(-0.97,-0.34) | 0.001 |  | -0.78(-0.98,-0.58) | <0.001 |  | -0.64(-0.92,-0.35) | 0.001 |
| Slovakia | -1.34(-1.49,-1.18) | <0.001 |  | -1.27(-1.71,-0.84) | <0.001 |  | -1.36(-1.50,-1.22) | <0.001 |  | -1.03(-1.48,-0.57) | 0.001 |
| Slovenia | -1.99(-2.15,-1.84) | <0.001 |  | -1.43(-1.98,-0.88) | <0.001 |  | -1.63(-1.77,-1.48) | <0.001 |  | -0.91(-1.43,-0.38) | 0.004 |
| **Eastern Europe** |  |  |  |  |  |  |  |  |  |  |  |
| Belarus | 0.26(-0.12,0.65) | 0.172 |  | -1.58(-2.36,-0.80) | 0.002 |  | 0.12(-0.30,0.54) | 0.561 |  | -1.97(-2.89,-1.03) | 0.001 |
| Estonia | -0.67(-0.84,-0.51) | <0.001 |  | -0.79(-1.17,-0.40) | 0.002 |  | -1.14(-1.35,-0.92) | <0.001 |  | -0.91(-1.17,-0.66) | <0.001 |
| Latvia | -0.94(-1.25,-0.62) | <0.001 |  | -1.92(-2.25,-1.59) | <0.001 |  | -1.03(-1.37,-0.70) | <0.001 |  | -1.82(-2.17,-1.48) | <0.001 |
| Lithuania | -0.31(-0.59,-0.03) | 0.033 |  | -1.64(-2.05,-1.22) | <0.001 |  | -0.42(-0.72,-0.11) | 0.010 |  | -1.78(-2.15,-1.40) | <0.001 |
| Moldova | -0.17(-0.44,0.09) | 0.187 |  | -0.98(-2.06,0.11) | 0.072 |  | -0.01(-0.28,0.26) | 0.953 |  | -1.37(-2.31,-0.42) | 0.010 |
| Russian Federation | -0.38(-0.90,0.14) | 0.147 |  | -2.26(-2.80,-1.72) | <0.001 |  | -0.37(-0.91,0.16) | 0.165 |  | -2.25(-2.77,-1.72) | <0.001 |
| Ukraine | 0.25(-0.06,0.56) | 0.114 |  | 0.78(-0.36,1.94) | 0.155 |  | 0.32(0.00,0.64) | 0.047 |  | 1.38(0.11,2.67) | 0.037 |
| **Western Europe** |  |  |  |  |  |  |  |  |  |  |  |
| Cyprus | -1.98(-2.20,-1.75) | <0.001 |  | -1.88(-2.10,-1.65) | <0.001 |  | -1.40(-1.64,-1.16) | <0.001 |  | -1.32(-1.73,-0.90) | <0.001 |
| Greece | -1.00(-1.12,-0.89) | <0.001 |  | -0.90(-1.42,-0.37) | 0.004 |  | -0.47(-0.56,-0.37) | <0.001 |  | -0.56(-0.94,-0.18) | 0.009 |
| Israel | -2.31(-2.51,-2.12) | <0.001 |  | -0.95(-1.46,-0.44) | 0.003 |  | -1.70(-1.85,-1.55) | <0.001 |  | -0.51(-0.94,-0.09) | 0.024 |

(DALYs, disability-adjusted life-years; BMI, Body Mass Index; BRI, Belt and Road Initiative.)
