# Supplementary material for: Spatial and temporal patterns of disease burden attributable to high BMI in Belt and Road Initiative countries, 1990–2019
Source: Public Health Nutr. 2024 Jun 5;27(1):e158. doi: 10.1017/S1368980024001253 (PMC11617424; doi:10.1017/S1368980024001253)
Supplement: Xu et al. supplementary material 4 — Xu et al. supplementary material [file S1368980024001253sup004.docx]

**Table S4** The average annual percentage change (AAPC) of mortality and DALY rates attributed to high BMI, stratified by gender for 1990-2019 in the BRI countries

|  | **Male** |  |  | **Female** |  |
| --- | --- | --- | --- | --- | --- |
| **Countries** | **AAPC *95%CI*** | ***P* value** |  | **AAPC *95%CI*** | ***P* value** |
| **East Asia** |  |  |  |  |  |
| China | 2.00(1.92,2.09) | <0.001 |  | 0.73(0.60,0.86) | <0.001 |
| **Central Asia** |  |  |  |  |  |
| Armenia | 1.71(1.43,1.98) | <0.001 |  | 0.07(-0.11,0.24) | 0.429 |
| Azerbaijan | 1.30(1.04,1.57) | <0.001 |  | 1.05(0.89,1.20) | <0.001 |
| Georgia | -0.31(-0.50,-0.12) | 0.002 |  | -0.79(-0.97,-0.60) | <0.001 |
| Kazakhstan | -0.26(-0.91,0.39) | 0.415 |  | -0.79(-1.28,-0.29) | 0.003 |
| Kyrgyzstan | -0.07(-0.43,0.30) | 0.707 |  | -0.73(-1.08,-0.37) | <0.001 |
| Mongolia | 1.02(0.67,1.37) | <0.001 |  | -1.25(-1.61,-0.89) | <0.001 |
| Tajikistan | 1.87(1.42,2.33) | <0.001 |  | 1.54(1.20,1.88) | <0.001 |
| Turkmenistan | 1.22(0.87,1.57) | <0.001 |  | 0.19(-0.07,0.46) | 0.150 |
| Uzbekistan | 2.30(1.89,2.71) | <0.001 |  | 1.98(1.56,2.41) | <0.001 |
| **South Asia** |  |  |  |  |  |
| Bangladesh | 4.61(4.20,5.01) | <0.001 |  | 4.26(3.82,4.70) | <0.001 |
| Bhutan | 3.08(2.94,3.22) | <0.001 |  | 1.75(1.54,1.95) | <0.001 |
| India | 3.25(3.15,3.36) | <0.001 |  | 2.52(2.38,2.67) | <0.001 |
| Nepal | 4.79(4.60,4.97) | <0.001 |  | 3.61(3.34,3.87) | <0.001 |
| Pakistan | 3.90(3.48,4.32) | <0.001 |  | 3.47(3.12,3.82) | <0.001 |
| **Southeast Asia** |  |  |  |  |  |
| Cambodia | 3.45(3.19,3.71) | <0.001 |  | 1.82(1.65,1.98) | <0.001 |
| Indonesia | 3.97(3.86,4.08) | <0.001 |  | 3.74(3.54,3.94) | <0.001 |
| Lao | 3.83(3.64,4.02) | <0.001 |  | 2.64(2.48,2.81) | <0.001 |
| Malaysia | 1.07(0.90,1.24) | <0.001 |  | 0.29(0.15,0.43) | <0.001 |
| Maldives | 0.85(0.64,1.05) | <0.001 |  | -0.31(-0.55,-0.06) | 0.018 |
| Burma | 3.09(2.80,3.38) | <0.001 |  | 2.48(2.31,2.65) | <0.001 |
| Philippines | 3.65(3.32,3.99) | <0.001 |  | 3.37(3.01,3.72) | <0.001 |
| Sri Lanka | 1.91(1.74,2.09) | <0.001 |  | 1.99(1.83,2.15) | <0.001 |
| Thailand | 1.56(1.24,1.88) | <0.001 |  | 1.06(0.73,1.39) | <0.001 |
| Viet Nam | 5.10(4.73,5.47) | <0.001 |  | 3.45(3.11,3.79) | <0.001 |
| **High-income Asia pacific** | |  |  |  |  |
| Brunei | 1.43(1.18,1.67) | <0.001 |  | 0.81(0.65,0.98) | <0.001 |
| Singapore | -0.50(-0.68,-0.32) | <0.001 |  | -0.82(-0.94,-0.69) | <0.001 |
| **North Africa and Middle East** | |  |  |  |  |
| Afghanistan | 1.78(1.21,2.35) | <0.001 |  | 1.07(0.63,1.51) | <0.001 |
| Bahrain | -1.06(-1.23,-0.88) | <0.001 |  | -0.90(-1.05,-0.74) | <0.001 |
| Egypt | 1.24(1.18,1.31) | <0.001 |  | 0.86(0.78,0.94) | <0.001 |
| Iran | 0.46(0.33,0.59) | <0.001 |  | -0.30(-0.46,-0.15) | <0.001 |
| Iraq | -0.51(-0.59,-0.44) | <0.001 |  | -0.90(-0.99,-0.81) | <0.001 |
| Jordan | -0.18(-0.27,-0.08) | 0.001 |  | -1.92(-2.30,-1.53) | <0.001 |
| Kuwait | 0.42(0.02,0.82) | 0.040 |  | -1.19(-1.54,-0.83) | <0.001 |
| Lebanon | 0.83(0.47,1.19) | <0.001 |  | -0.39(-0.55,-0.24) | <0.001 |
| Oman | 1.49(0.95,2.04) | <0.001 |  | 0.86(0.50,1.21) | <0.001 |
| Palestine | -0.07(-0.26,0.13) | 0.490 |  | -0.26(-0.48,-0.04) | 0.021 |
| Qatar | -0.58(-0.82,-0.33) | <0.001 |  | 0.28(-0.15,0.70) | 0.193 |
| Saudi Arabia | 1.45(1.19,1.72) | <0.001 |  | 0.44(0.16,0.72) | 0.003 |
| Syrian Arab Republic | -0.15(-0.34,0.05) | 0.133 |  | -0.78(-1.00,-0.55) | <0.001 |
| Turkey | -0.90(-1.10,-0.71) | <0.001 |  | -1.08(-1.35,-0.81) | <0.001 |
| United Arab Emirates | 0.04(-0.14,0.23) | 0.621 |  | -0.27(-1.06,0.52) | 0.482 |
| Yemen | 1.81(1.71,1.92) | <0.001 |  | 1.30(1.13,1.46) | <0.001 |
| **Central Europe** |  |  |  |  |  |
| Albania | 0.60(0.37,0.83) | <0.001 |  | 0.38(0.22,0.55) | <0.001 |
| Bosnia and Herzegovina | 1.28(1.08,1.47) | <0.001 |  | 0.33(0.17,0.48) | <0.001 |
| Bulgaria | -0.65(-0.89,-0.41) | <0.001 |  | -1.02(-1.24,-0.80) | <0.001 |
| Croatia | -0.75(-0.84,-0.67) | <0.001 |  | -1.07(-1.14,-1.00) | <0.001 |
| Czechia | -1.65(-1.78,-1.52) | <0.001 |  | -1.18(-1.30,-1.06) | <0.001 |
| Hungary | -1.45(-1.56,-1.34) | <0.001 |  | -1.43(-1.52,-1.33) | <0.001 |
| Montenegro | 0.21(0.08,0.35) | 0.003 |  | 0.32(0.10,0.53) | 0.005 |
| Macedonia | 0.26(0.06,0.45) | 0.012 |  | -0.20(-0.35,-0.06) | 0.009 |
| Poland | -1.53(-1.67,-1.40) | <0.001 |  | -1.65(-1.77,-1.53) | <0.001 |
| Romania | -0.45(-0.74,-0.16) | 0.004 |  | -1.19(-1.37,-1.01) | <0.001 |
| Serbia | -0.46(-0.66,-0.25) | <0.001 |  | -1.11(-1.32,-0.90) | <0.001 |
| Slovakia | -1.39(-1.57,-1.20) | <0.001 |  | -1.35(-1.46,-1.24) | <0.001 |
| Slovenia | -1.63(-1.83,-1.43) | <0.001 |  | -1.68(-1.80,-1.56) | <0.001 |
| **Eastern Europe** |  |  |  |  |  |
| Belarus | 0.83(0.34,1.33) | 0.002 |  | -0.53(-0.89,-0.17) | 0.005 |
| Estonia | -1.17(-1.46,-0.87) | <0.001 |  | -1.09(-1.25,-0.93) | <0.001 |
| Latvia | -0.99(-1.43,-0.54) | <0.001 |  | -1.06(-1.30,-0.82) | <0.001 |
| Lithuania | 0.02(-0.37,0.41) | 0.911 |  | -0.88(-1.10,-0.66) | <0.001 |
| Moldova | 0.95(0.63,1.26) | <0.001 |  | -0.77(-1.04,-0.51) | <0.001 |
| Russian Federation | -0.02(-0.64,0.61) | 0.958 |  | -0.71(-1.16,-0.26) | 0.003 |
| Ukraine | 0.93(0.56,1.31) | <0.001 |  | -0.25(-0.52,0.03) | 0.078 |
| **Western Europe** |  |  |  |  |  |
| Cyprus | -1.25(-1.54,-0.97) | <0.001 |  | -1.62(-1.81,-1.43) | <0.001 |
| Greece | -0.27(-0.41,-0.13) | <0.001 |  | -0.68(-0.75,-0.61) | <0.001 |
| Israel | -1.75(-1.91,-1.58) | <0.001 |  | -1.67(-1.80,-1.54) | <0.001 |

(DALYs, disability-adjusted life-years; BMI, Body Mass Index; BRI, Belt and Road Initiative.)
