# Supplementary material for: Spatial and temporal patterns of disease burden attributable to high BMI in Belt and Road Initiative countries, 1990–2019
Source: Public Health Nutr. 2024 Jun 5;27(1):e158. doi: 10.1017/S1368980024001253 (PMC11617424; doi:10.1017/S1368980024001253)
Supplement: Xu et al. supplementary material 5 — Xu et al. supplementary material [file S1368980024001253sup005.docx]

**Table S5** The average annual percentage change (AAPC) of DALY rates attributed to high BMI, stratified by age for 1990-2019 in the BRI countries

|  | **20-54 yrs** | | **50-74 yrs** | | **≥75 yrs** | |
| --- | --- | --- | --- | --- | --- | --- |
| **Countries** | **AAPC *95%CI*** | ***P* value** | **AAPC *95%CI*** | ***P* value** | **AAPC *95%CI*** | ***P* value** |
| **East Asia** |  |  |  |  |  |  |
| China | 1.32(1.05,1.58) | <0.001 | 0.58(0.40,0.76) | <0.001 | 1.43(1.28,1.58) | <0.001 |
| **Central Asia** |  |  |  |  |  |  |
| Armenia | 1.38(0.97,1.79) | <0.001 | -0.68(-1.11,-0.26) | 0.003 | 1.03(0.88,1.19) | <0.001 |
| Azerbaijan | 1.04(0.76,1.32) | <0.001 | 0.04(-0.32,0.41) | 0.806 | 3.16(2.88,3.44) | <0.001 |
| Georgia | -0.81(-1.17,-0.45) | <0.001 | -1.10(-1.39,-0.80) | <0.001 | 0.37(0.05,0.70) | 0.027 |
| Kazakhstan | -0.70(-1.34,-0.06) | 0.034 | -1.23(-1.84,-0.63) | <0.001 | -0.06(-0.42,0.30) | 0.734 |
| Kyrgyzstan | -0.96(-1.36,-0.56) | <0.001 | -1.43(-1.90,-0.95) | <0.001 | 1.13(0.76,1.51) | <0.001 |
| Mongolia | -0.22(-0.50,0.06) | 0.113 | -1.66(-2.07,-1.24) | <0.001 | -0.54(-0.94,-0.14) | 0.01 |
| Tajikistan | 1.21(0.67,1.76) | <0.001 | 0.81(0.55,1.06) | <0.001 | 3.34(3.07,3.61) | <0.001 |
| Turkmenistan | 1.55(1.15,1.94) | <0.001 | -0.31(-0.61,-0.01) | 0.045 | 0.24(-0.01,0.50) | 0.061 |
| Uzbekistan | 1.94(1.58,2.31) | <0.001 | 0.90(0.33,1.47) | 0.003 | 3.97(3.42,4.53) | <0.001 |
| **South Asia** |  |  |  |  |  |  |
| Bangladesh | 4.91(4.40,5.43) | <0.001 | 4.57(4.09,5.05) | <0.001 | 2.99(2.52,3.46) | <0.001 |
| Bhutan | 1.31(1.03,1.59) | <0.001 | 1.62(1.41,1.84) | <0.001 | 3.47(3.31,3.62) | <0.001 |
| India | 2.59(2.42,2.75) | <0.001 | 2.92(2.79,3.06) | <0.001 | 2.08(1.62,2.53) | <0.001 |
| Nepal | 3.67(3.20,4.14) | <0.001 | 3.53(3.22,3.83) | <0.001 | 4.16(3.97,4.36) | <0.001 |
| Pakistan | 3.37(3.09,3.66) | <0.001 | 3.45(3.10,3.81) | <0.001 | 3.61(3.22,4.00) | <0.001 |
| **Southeast Asia** |  |  |  |  |  |  |
| Cambodia | 1.54(1.43,1.65) | <0.001 | 1.69(1.53,1.86) | <0.001 | 3.48(3.31,3.65) | <0.001 |
| Indonesia | 3.85(3.56,4.14) | <0.001 | 4.53(4.25,4.80) | <0.001 | 3.64(3.44,3.84) | <0.001 |
| Lao | 2.74(2.52,2.96) | <0.001 | 2.55(2.39,2.71) | <0.001 | 2.84(2.74,2.93) | <0.001 |
| Malaysia | 0.77(0.65,0.88) | <0.001 | -0.03(-0.20,0.14) | 0.712 | 1.74(1.44,2.04) | <0.001 |
| Maldives | -0.10(-0.42,0.23) | 0.539 | -0.58(-0.83,-0.34) | <0.001 | 0.84(0.63,1.05) | <0.001 |
| Burma | 2.51(2.21,2.82) | <0.001 | 2.69(2.52,2.86) | <0.001 | 3.48(3.22,3.74) | <0.001 |
| Philippines | 4.35(3.83,4.87) | <0.001 | 3.39(3.03,3.76) | <0.001 | 2.59(2.40,2.78) | <0.001 |
| Sri Lanka | 2.10(1.97,2.23) | <0.001 | 1.91(1.77,2.05) | <0.001 | 3.37(3.11,3.63) | <0.001 |
| Thailand | 1.86(1.41,2.31) | <0.001 | 0.97(0.67,1.28) | <0.001 | 2.34(2.07,2.62) | <0.001 |
| Viet Nam | 4.20(3.82,4.59) | <0.001 | 3.01(2.74,3.28) | <0.001 | 4.09(3.81,4.36) | <0.001 |
| **High-income Asia pacific** |  |  |  |  |  |  |
| Brunei | 1.81(1.46,2.16) | <0.001 | 0.56(0.46,0.65) | <0.001 | 1.13(0.80,1.47) | <0.001 |
| Singapore | 0.82(0.69,0.95) | <0.001 | -1.39(-1.55,-1.23) | <0.001 | 0.13(0.02,0.25) | 0.023 |
| **North Africa and Middle East** | |  |  |  |  |  |
| Afghanistan | 0.66(0.03,1.29) | 0.040 | 0.98(0.58,1.38) | <0.001 | 2.23(1.58,2.88) | <0.001 |
| Bahrain | -0.11(-0.39,0.16) | 0.402 | -2.22(-2.48,-1.95) | <0.001 | 0.58(0.27,0.90) | 0.001 |
| Egypt | -0.04(-0.18,0.11) | 0.609 | 0.78(0.70,0.87) | <0.001 | 1.52(1.39,1.65) | <0.001 |
| Iran | -0.33(-0.51,-0.14) | 0.001 | -0.89(-1.13,-0.65) | <0.001 | 0.40(0.22,0.57) | <0.001 |
| Iraq | -1.17(-1.41,-0.94) | <0.001 | -1.11(-1.19,-1.03) | <0.001 | 0.18(-0.01,0.37) | 0.064 |
| Jordan | -1.54(-1.75,-1.32) | <0.001 | -2.24(-2.73,-1.74) | <0.001 | -1.04(-1.40,-0.68) | <0.001 |
| Kuwait | -0.69(-0.98,-0.39) | <0.001 | -1.98(-2.51,-1.45) | <0.001 | -0.24(-0.50,0.02) | 0.07 |
| Lebanon | -0.53(-0.81,-0.26) | <0.001 | -0.40(-0.51,-0.29) | <0.001 | -0.14(-0.21,-0.07) | <0.001 |
| Oman | -0.74(-0.99,-0.49) | <0.001 | 0.19(-0.25,0.63) | 0.392 | 3.22(2.92,3.51) | <0.001 |
| Palestine | -0.58(-0.87,-0.30) | <0.001 | -0.85(-0.99,-0.72) | <0.001 | 1.23(0.85,1.61) | <0.001 |
| Qatar | -1.39(-1.61,-1.17) | <0.001 | -1.63(-2.12,-1.14) | <0.001 | 2.63(1.95,3.30) | <0.001 |
| Saudi Arabia | 0.79(0.63,0.96) | <0.001 | 0.05(-0.31,0.41) | 0.760 | 0.75(0.46,1.04) | <0.001 |
| Syrian Arab Republic | -0.38(-0.88,0.12) | 0.133 | -1.09(-1.37,-0.80) | <0.001 | -0.17(-0.54,0.21) | 0.375 |
| Turkey | -0.64(-0.81,-0.48) | <0.001 | -1.57(-1.81,-1.33) | <0.001 | 0.30(-0.06,0.66) | 0.1 |
| United Arab Emirates | 0.19(-0.55,0.95) | 0.601 | -1.44(-1.79,-1.08) | <0.001 | 0.55(-0.93,2.05) | 0.457 |
| Yemen | 1.10(0.88,1.32) | <0.001 | 1.05(0.88,1.22) | <0.001 | 2.01(1.79,2.24) | <0.001 |
| **Central Europe** |  |  |  |  |  |  |
| Albania | 1.89(1.56,2.22) | <0.001 | 0.16(0.01,0.31) | 0.033 | 0.66(0.48,0.84) | <0.001 |
| Bosnia and Herzegovina | 0.76(0.63,0.90) | <0.001 | -0.09(-0.33,0.15) | 0.441 | 1.58(1.22,1.93) | <0.001 |
| Bulgaria | -0.51(-0.84,-0.18) | 0.004 | -1.08(-1.34,-0.81) | <0.001 | -0.76(-0.96,-0.55) | <0.001 |
| Croatia | -0.44(-0.55,-0.32) | <0.001 | -1.69(-1.92,-1.46) | <0.001 | -0.13(-0.26,0.01) | 0.075 |
| Czechia | -0.66(-0.89,-0.44) | <0.001 | -1.51(-1.80,-1.21) | <0.001 | -0.46(-0.54,-0.39) | <0.001 |
| Hungary | -1.80(-1.96,-1.65) | <0.001 | -1.47(-1.66,-1.29) | <0.001 | -0.55(-0.63,-0.48) | <0.001 |
| Montenegro | 0.42(0.06,0.79) | 0.025 | 0.29(-0.05,0.62) | 0.089 | 1.14(0.95,1.33) | <0.001 |
| Macedonia | 0.12(-0.12,0.37) | 0.308 | -0.56(-0.80,-0.31) | <0.001 | 0.45(0.33,0.57) | <0.001 |
| Poland | -1.46(-1.61,-1.31) | <0.001 | -2.04(-2.30,-1.78) | <0.001 | -1.26(-1.39,-1.13) | <0.001 |
| Romania | -0.56(-0.76,-0.35) | <0.001 | -1.47(-1.75,-1.19) | <0.001 | -0.63(-0.78,-0.48) | <0.001 |
| Serbia | -1.08(-1.32,-0.85) | <0.001 | -1.44(-1.83,-1.05) | <0.001 | -0.34(-0.55,-0.13) | 0.003 |
| Slovakia | -0.80(-0.94,-0.66) | <0.001 | -1.97(-2.18,-1.76) | <0.001 | -0.36(-0.52,-0.21) | <0.001 |
| Slovenia | -0.38(-0.53,-0.23) | <0.001 | -2.31(-2.53,-2.09) | <0.001 | -1.08(-1.22,-0.93) | <0.001 |
| **Eastern Europe** |  |  |  |  |  |  |
| Belarus | -0.08(-0.53,0.38) | 0.738 | -1.16(-1.71,-0.60) | <0.001 | 0.34(0.11,0.58) | 0.005 |
| Estonia | -1.74(-2.08,-1.40) | <0.001 | -1.67(-1.92,-1.42) | <0.001 | 0.76(0.67,0.85) | <0.001 |
| Latvia | -1.29(-1.65,-0.94) | <0.001 | -1.21(-1.50,-0.91) | <0.001 | -0.11(-0.24,0.01) | 0.072 |
| Lithuania | -0.54(-0.87,-0.21) | 0.002 | -1.13(-1.42,-0.84) | <0.001 | -0.14(-0.26,-0.03) | 0.018 |
| Moldova | -0.64(-1.07,-0.21) | 0.005 | -1.14(-1.48,-0.80) | <0.001 | -0.07(-0.23,0.09) | 0.398 |
| Russian Federation | -0.37(-1.03,0.29) | 0.259 | -1.34(-1.97,-0.71) | <0.001 | 0.09(-0.31,0.50) | 0.643 |
| Ukraine | -0.01(-0.32,0.29) | 0.921 | -0.65(-1.09,-0.20) | 0.006 | 0.01(-0.25,0.27) | 0.914 |
| **Western Europe** |  |  |  |  |  |  |
| Cyprus | -0.48(-0.73,-0.23) | 0.001 | -1.80(-2.07,-1.53) | <0.001 | -1.73(-1.84,-1.62) | <0.001 |
| Greece | 0.76(0.70,0.81) | <0.001 | -1.06(-1.17,-0.94) | <0.001 | -0.77(-0.91,-0.64) | <0.001 |
| Israel | -0.44(-0.60,-0.28) | <0.001 | -2.32(-2.57,-2.08) | <0.001 | -1.05(-1.21,-0.90) | <0.001 |

(DALYs, disability-adjusted life-years; BMI, Body Mass Index; BRI, Belt and Road Initiative.)
