# Supplementary material for: Spatial and temporal patterns of disease burden attributable to high BMI in Belt and Road Initiative countries, 1990–2019
Source: Public Health Nutr. 2024 Jun 5;27(1):e158. doi: 10.1017/S1368980024001253 (PMC11617424; doi:10.1017/S1368980024001253)
Supplement: Xu et al. supplementary material 6 — Xu et al. supplementary material [file S1368980024001253sup006.docx]

**Table S6** The average annual percentage change (AAPC) of age-standardized rates for DALYs attributed to high BMI, stratified by disease for 1990-2019 in the BRI countries

|  | **Cardiovascular diseases** | | **Chronic respiratory diseases** | | **Diabetes and kidney diseases** | | **Digestive diseases** | | **Musculoskeletal disorders** | | **Neoplasms** | | **Neurological disorders** | | **Sense organ diseases** | |
| --- | --- | --- | --- | --- | --- | --- | --- | --- | --- | --- | --- | --- | --- | --- | --- | --- |
| **Countries** | **AAPC *95%CI*** | ***P* value** | **AAPC *95%CI*** | ***P* value** | **AAPC *95%CI*** | ***P* value** | **AAPC *95%CI*** | ***P* value** | **AAPC *95%CI*** | ***P* value** | **AAPC *95%CI*** | ***P* value** | **AAPC *95%CI*** | ***P* value** | **AAPC *95%CI*** | ***P* value** |
| **East Asia** |  |  |  |  |  |  |  |  |  |  |  |  |  |  |  |  |
| China | 1.06(0.98,1.14) | <0.001 | -0.78(-1.13,-0.43) | <0.001 | 2.99(2.77,3.22) | <0.001 | 0.82(0.67,0.96) | <0.001 | 2.87(2.68,3.07) | <0.001 | 0.66(0.48,0.84) | <0.001 | 2.81(2.75,2.88) | <0.001 | 2.87(2.49,3.25) | <0.001 |
| **Central Asia** |  |  |  |  |  |  |  |  |  |  |  |  |  |  |  |  |
| Armenia | -0.02(-0.23,0.20) | 0.883 | -0.35(-0.62,-0.08) | 0.012 | 2.39(1.94,2.84) | <0.001 | 1.28(0.97,1.59) | <0.001 | 2.04(1.89,2.18) | <0.001 | 3.08(2.67,3.49) | <0.001 | 2.63(2.44,2.82) | <0.001 | 1.40(1.26,1.54) | <0.001 |
| Azerbaijan | 0.83(0.64,1.02) | <0.001 | -1.32(-1.89,-0.74) | <0.001 | 2.78(2.59,2.97) | <0.001 | -0.44(-0.69,-0.19) | 0.001 | 1.69(1.39,1.98) | <0.001 | 1.71(1.34,2.07) | <0.001 | 1.92(1.63,2.20) | <0.001 | 1.01(0.77,1.25) | <0.001 |
| Georgia | -1.36(-1.61,-1.12) | <0.001 | -2.57(-2.97,-2.18) | <0.001 | 3.27(3.01,3.52) | <0.001 | 0.89(0.60,1.18) | <0.001 | 0.33(0.24,0.43) | <0.001 | 1.56(1.20,1.93) | <0.001 | 0.36(0.28,0.44) | <0.001 | 0.00(-0.09,0.10) | 0.952 |
| Kazakhstan | -1.07(-1.82,-0.32) | 0.007 | -1.25(-1.73,-0.77) | <0.001 | 1.93(1.64,2.22) | <0.001 | -1.38(-1.69,-1.06) | <0.001 | 0.90(0.80,1.00) | <0.001 | -0.88(-1.04,-0.71) | <0.001 | 0.64(0.55,0.72) | <0.001 | 0.07(0.01,0.14) | 0.028 |
| Kyrgyzstan | -0.52(-0.95,-0.08) | 0.021 | -3.48(-3.78,-3.17) | <0.001 | 0.22(-0.03,0.47) | 0.085 | -1.22(-1.36,-1.07) | <0.001 | 0.65(0.56,0.74) | <0.001 | -0.52(-0.72,-0.31) | <0.001 | 0.74(0.69,0.78) | <0.001 | 0.02(-0.04,0.08) | 0.54 |
| Mongolia | -0.27(-0.69,0.15) | 0.197 | -2.73(-3.05,-2.41) | <0.001 | -0.66(-0.91,-0.40) | <0.001 | -1.96(-2.18,-1.74) | <0.001 | 0.96(0.89,1.03) | <0.001 | 1.82(1.59,2.04) | <0.001 | 0.83(0.75,0.90) | <0.001 | -0.09(-0.22,0.04) | 0.185 |
| Tajikistan | 1.29(0.95,1.64) | <0.001 | -1.11(-1.57,-0.64) | <0.001 | 3.95(3.35,4.55) | <0.001 | 0.30(-0.10,0.71) | 0.138 | 0.92(0.55,1.30) | <0.001 | 0.71(0.27,1.14) | 0.002 | 1.06(0.70,1.43) | <0.001 | 0.33(0.00,0.67) | 0.053 |
| Turkmenistan | 0.59(0.21,0.98) | 0.004 | -4.52(-5.13,-3.91) | <0.001 | 2.13(1.90,2.35) | <0.001 | -0.04(-0.12,0.05) | 0.395 | 1.56(1.48,1.63) | <0.001 | -0.73(-1.44,-0.01) | 0.046 | 1.31(1.27,1.35) | <0.001 | 0.50(0.38,0.62) | <0.001 |
| Uzbekistan | 2.09(1.61,2.57) | <0.001 | -1.96(-2.37,-1.55) | <0.001 | 3.43(2.97,3.89) | <0.001 | 0.60(0.52,0.69) | <0.001 | 1.20(1.10,1.29) | <0.001 | 0.28(0.03,0.53) | 0.028 | 1.36(1.31,1.42) | <0.001 | 0.42(0.33,0.51) | <0.001 |
| **South Asia** |  |  |  |  |  |  |  |  |  |  |  |  |  |  |  |  |
| Bangladesh | 4.36(3.90,4.82) | <0.001 | 0.18(-0.07,0.42) | 0.149 | 5.38(4.97,5.79) | <0.001 | 3.28(2.96,3.59) | <0.001 | 5.05(4.81,5.30) | <0.001 | 3.96(3.74,4.18) | <0.001 | 3.68(3.58,3.78) | <0.001 | 3.56(3.26,3.86) | <0.001 |
| Bhutan | 1.63(1.46,1.81) | <0.001 | -0.46(-0.59,-0.32) | <0.001 | 3.86(3.67,4.05) | <0.001 | 1.19(1.06,1.33) | <0.001 | 3.31(3.05,3.56) | <0.001 | 3.18(3.03,3.33) | <0.001 | 3.51(3.37,3.66) | <0.001 | 0.85(0.71,0.99) | <0.001 |
| India | 2.51(2.41,2.61) | <0.001 | 0.77(0.63,0.92) | <0.001 | 3.96(3.77,4.16) | <0.001 | 3.51(3.23,3.79) | <0.001 | 2.94(2.85,3.04) | <0.001 | 3.64(3.47,3.81) | <0.001 | 3.85(3.63,4.06) | <0.001 | 2.54(2.30,2.79) | <0.001 |
| Nepal | 3.68(3.45,3.92) | <0.001 | 2.30(2.07,2.53) | <0.001 | 5.96(5.82,6.11) | <0.001 | 2.85(2.56,3.15) | <0.001 | 5.16(5.01,5.30) | <0.001 | 5.16(4.85,5.47) | <0.001 | 4.90(4.73,5.07) | <0.001 | 3.45(3.37,3.52) | <0.001 |
| Pakistan | 3.18(2.76,3.59) | <0.001 | 0.87(0.47,1.27) | <0.001 | 5.29(4.87,5.71) | <0.001 | 1.88(1.67,2.09) | <0.001 | 3.40(3.16,3.63) | <0.001 | 3.92(3.52,4.33) | <0.001 | 3.25(3.02,3.48) | <0.001 | 2.44(2.08,2.80) | <0.001 |
| **Southeast Asia** |  |  |  |  |  |  |  |  |  |  |  |  |  |  |  |  |
| Cambodia | 1.97(1.81,2.14) | <0.001 | 1.03(0.86,1.20) | <0.001 | 3.61(3.34,3.89) | <0.001 | 1.74(1.64,1.83) | <0.001 | 3.17(3.00,3.35) | <0.001 | 3.15(2.95,3.34) | <0.001 | 3.92(3.69,4.14) | <0.001 | 1.26(1.15,1.38) | <0.001 |
| Indonesia | 3.51(3.34,3.68) | <0.001 | 1.94(1.78,2.11) | <0.001 | 4.80(4.67,4.93) | <0.001 | 2.37(2.26,2.47) | <0.001 | 3.97(3.79,4.16) | <0.001 | 4.67(4.48,4.85) | <0.001 | 3.25(3.02,3.47) | <0.001 | 2.44(2.32,2.56) | <0.001 |
| Lao | 3.09(2.88,3.29) | <0.001 | 0.34(0.29,0.39) | <0.001 | 3.71(3.59,3.83) | <0.001 | 2.49(2.41,2.58) | <0.001 | 4.03(3.88,4.18) | <0.001 | 3.05(2.96,3.15) | <0.001 | 3.50(3.40,3.59) | <0.001 | 2.96(2.86,3.06) | <0.001 |
| Malaysia | 0.46(0.32,0.59) | <0.001 | -2.91(-3.44,-2.37) | <0.001 | 0.95(0.71,1.20) | <0.001 | 1.46(1.26,1.66) | <0.001 | 2.01(1.88,2.14) | <0.001 | 2.12(2.02,2.23) | <0.001 | 2.45(2.33,2.56) | <0.001 | 0.80(0.70,0.90) | <0.001 |
| Maldives | -0.83(-1.05,-0.61) | <0.001 | -2.38(-2.68,-2.09) | <0.001 | 1.79(1.52,2.06) | <0.001 | 2.00(1.86,2.13) | <0.001 | 4.24(4.03,4.44) | <0.001 | 1.90(1.74,2.06) | <0.001 | 3.64(3.56,3.71) | <0.001 | 2.06(1.92,2.20) | <0.001 |
| Burma | 2.25(2.11,2.40) | <0.001 | 0.49(0.35,0.64) | <0.001 | 3.98(3.76,4.20) | <0.001 | 3.29(3.05,3.52) | <0.001 | 4.72(4.43,5.01) | <0.001 | 3.32(3.14,3.50) | <0.001 | 4.32(4.02,4.62) | <0.001 | 3.00(2.80,3.20) | <0.001 |
| Philippines | 5.07(4.31,5.83) | <0.001 | 0.15(0.02,0.27) | 0.028 | 2.51(2.37,2.66) | <0.001 | 1.36(1.20,1.52) | <0.001 | 1.77(1.63,1.90) | <0.001 | 1.07(0.75,1.39) | <0.001 | 1.46(1.26,1.67) | <0.001 | 1.36(1.28,1.44) | <0.001 |
| Sri Lanka | 0.22(0.03,0.42) | 0.028 | -0.15(-0.28,-0.02) | 0.021 | 4.56(4.21,4.92) | <0.001 | 1.25(1.13,1.36) | <0.001 | 2.11(2.07,2.16) | <0.001 | 2.00(1.75,2.25) | <0.001 | 2.78(2.67,2.89) | <0.001 | 1.80(1.70,1.89) | <0.001 |
| Thailand | 0.49(0.14,0.84) | 0.007 | -0.61(-0.87,-0.35) | <0.001 | 1.90(1.60,2.20) | <0.001 | 0.42(0.30,0.54) | <0.001 | 3.31(3.15,3.47) | <0.001 | 2.70(2.39,3.02) | <0.001 | 3.19(2.95,3.43) | <0.001 | 2.02(1.86,2.19) | <0.001 |
| Viet Nam | 3.97(3.63,4.32) | <0.001 | 2.25(1.95,2.54) | <0.001 | 5.01(4.54,5.49) | <0.001 | 2.04(1.82,2.25) | <0.001 | 4.46(4.22,4.70) | <0.001 | 5.41(5.06,5.77) | <0.001 | 4.69(4.45,4.93) | <0.001 | 3.03(2.85,3.21) | <0.001 |
| **High-income Asia pacific** |  |  |  |  |  |  |  |  |  |  |  |  |  |  |  |  |
| Brunei | -0.15(-0.27,-0.03) | 0.014 | -0.76(-0.82,-0.69) | <0.001 | 1.91(1.60,2.23) | <0.001 | 1.62(1.51,1.72) | <0.001 | 2.38(2.23,2.54) | <0.001 | 2.77(2.51,3.04) | <0.001 | 2.42(2.32,2.52) | <0.001 | 1.99(1.89,2.10) | <0.001 |
| Singapore | -1.87(-2.02,-1.72) | <0.001 | -2.51(-2.90,-2.12) | <0.001 | 0.44(0.17,0.71) | 0.002 | 0.91(0.74,1.09) | <0.001 | 2.11(1.95,2.26) | <0.001 | 0.68(0.40,0.95) | <0.001 | 2.27(2.21,2.32) | <0.001 | 1.80(1.67,1.93) | <0.001 |
| **North Africa and Middle East** | |  |  |  |  |  |  |  |  |  |  |  |  |  |  |  |
| Afghanistan | 0.86(0.46,1.27) | <0.001 | 0.05(-0.28,0.38) | 0.760 | 3.04(2.42,3.66) | <0.001 | 1.86(1.34,2.38) | <0.001 | 2.26(1.58,2.95) | <0.001 | 1.96(1.35,2.58) | <0.001 | 2.41(1.68,3.14) | <0.001 | 2.48(1.94,3.01) | <0.001 |
| Bahrain | -3.70(-3.92,-3.48) | <0.001 | -2.84(-3.03,-2.65) | <0.001 | 1.59(1.25,1.92) | <0.001 | -2.62(-2.94,-2.30) | <0.001 | 0.92(0.87,0.98) | <0.001 | -0.77(-1.01,-0.53) | <0.001 | 0.80(0.71,0.88) | <0.001 | -0.35(-0.43,-0.27) | <0.001 |
| Egypt | 0.61(0.54,0.68) | <0.001 | -0.89(-0.96,-0.82) | <0.001 | 2.23(2.16,2.29) | <0.001 | 0.03(-0.04,0.11) | 0.343 | 1.38(1.29,1.48) | <0.001 | 2.54(2.32,2.75) | <0.001 | 1.10(0.99,1.21) | <0.001 | -0.06(-0.11,-0.02) | 0.01 |
| Iran | -0.94(-1.13,-0.75) | <0.001 | -1.86(-2.07,-1.64) | <0.001 | 2.53(2.44,2.62) | <0.001 | 1.10(0.85,1.34) | <0.001 | 1.48(1.42,1.54) | <0.001 | 1.38(1.25,1.50) | <0.001 | 1.80(1.68,1.91) | <0.001 | 0.79(0.69,0.89) | <0.001 |
| Iraq | -1.08(-1.19,-0.98) | <0.001 | -2.93(-3.09,-2.77) | <0.001 | 0.01(-0.04,0.06) | 0.726 | -1.10(-1.17,-1.02) | <0.001 | 0.46(0.34,0.57) | <0.001 | 1.08(0.73,1.43) | <0.001 | 0.82(0.59,1.05) | <0.001 | -0.70(-0.83,-0.58) | <0.001 |
| Jordan | -1.91(-2.15,-1.67) | <0.001 | -1.87(-2.03,-1.70) | <0.001 | -0.29(-0.57,0.00) | 0.051 | -1.20(-1.30,-1.11) | <0.001 | 1.48(1.43,1.54) | <0.001 | 0.77(0.66,0.88) | <0.001 | 1.20(1.17,1.24) | <0.001 | 0.32(0.24,0.39) | <0.001 |
| Kuwait | -0.72(-1.11,-0.32) | 0.001 | -0.99(-1.24,-0.74) | <0.001 | 0.13(-0.35,0.61) | 0.585 | 0.36(0.18,0.54) | <0.001 | 1.37(1.25,1.48) | <0.001 | 1.36(0.94,1.78) | <0.001 | 1.28(1.16,1.40) | <0.001 | 0.52(0.43,0.61) | <0.001 |
| Lebanon | -0.24(-0.55,0.07) | 0.118 | -1.23(-1.37,-1.09) | <0.001 | 1.09(0.91,1.26) | <0.001 | 0.54(0.37,0.70) | <0.001 | 1.34(1.26,1.43) | <0.001 | 2.01(1.82,2.20) | <0.001 | 1.20(1.14,1.26) | <0.001 | -0.25(-0.34,-0.15) | <0.001 |
| Oman | 0.44(-0.08,0.95) | 0.093 | 2.75(2.64,2.87) | <0.001 | 2.50(2.10,2.90) | <0.001 | 1.11(0.89,1.32) | <0.001 | 3.13(2.90,3.37) | <0.001 | 3.65(3.07,4.23) | <0.001 | 3.44(3.13,3.76) | <0.001 | 3.09(2.66,3.52) | <0.001 |
| Palestine | -0.92(-1.11,-0.73) | <0.001 | -1.71(-1.90,-1.52) | <0.001 | 1.09(0.88,1.31) | <0.001 | -0.02(-0.21,0.16) | 0.79 | 0.71(0.58,0.85) | <0.001 | 0.89(0.58,1.20) | <0.001 | 0.97(0.75,1.19) | <0.001 | -0.31(-0.38,-0.24) | <0.001 |
| Qatar | -2.15(-2.42,-1.89) | <0.001 | -0.77(-0.96,-0.58) | <0.001 | 1.08(0.60,1.56) | <0.001 | 0.04(-0.11,0.18) | 0.622 | 1.25(1.21,1.29) | <0.001 | 0.90(0.64,1.16) | <0.001 | 1.44(1.35,1.53) | <0.001 | -0.44(-0.58,-0.30) | <0.001 |
| Saudi Arabia | 0.88(0.56,1.20) | <0.001 | -0.76(-0.93,-0.59) | <0.001 | 1.26(1.01,1.50) | <0.001 | -1.00(-1.19,-0.80) | <0.001 | 1.93(1.79,2.08) | <0.001 | 1.97(1.63,2.30) | <0.001 | 1.86(1.70,2.02) | <0.001 | -0.77(-0.85,-0.69) | <0.001 |
| Syrian Arab Republic | -0.73(-0.95,-0.51) | <0.001 | -1.25(-1.43,-1.07) | <0.001 | 0.12(-0.12,0.36) | 0.308 | -0.54(-0.69,-0.39) | <0.001 | 1.01(0.92,1.09) | <0.001 | 0.08(-0.13,0.29) | 0.435 | 1.22(1.14,1.31) | <0.001 | -0.28(-0.33,-0.22) | <0.001 |
| Turkey | -1.87(-2.12,-1.63) | <0.001 | -1.46(-1.74,-1.18) | <0.001 | -0.02(-0.36,0.31) | 0.885 | -0.14(-0.30,0.03) | 0.094 | 0.99(0.95,1.03) | <0.001 | 0.28(-0.04,0.61) | 0.087 | 1.00(0.93,1.07) | <0.001 | -0.18(-0.28,-0.08) | 0.001 |
| United Arab Emirates | -0.61(-0.93,-0.29) | 0.001 | -1.44(-1.68,-1.19) | <0.001 | 0.63(0.14,1.12) | 0.013 | -0.01(-0.44,0.42) | 0.95 | 1.55(1.45,1.65) | <0.001 | 1.60(1.21,1.98) | <0.001 | 1.50(1.36,1.64) | <0.001 | 0.52(0.36,0.67) | <0.001 |
| Yemen | 1.23(1.10,1.35) | <0.001 | -0.31(-0.44,-0.18) | <0.001 | 3.14(2.94,3.34) | <0.001 | 2.20(1.99,2.40) | <0.001 | 2.47(2.30,2.65) | <0.001 | 2.66(2.45,2.88) | <0.001 | 2.32(2.09,2.55) | <0.001 | 1.69(1.57,1.82) | <0.001 |
| **Central Europe** |  |  |  |  |  |  |  |  |  |  |  |  |  |  |  |  |
| Albania | 0.29(0.07,0.51) | 0.012 | -2.02(-2.33,-1.70) | <0.001 | 1.26(1.11,1.41) | <0.001 | 0.66(0.53,0.79) | <0.001 | 1.51(1.41,1.60) | <0.001 | 0.82(0.61,1.04) | <0.001 | 1.04(0.97,1.10) | <0.001 | 0.67(0.60,0.73) | <0.001 |
| Bosnia and Herzegovina | -0.60(-0.73,-0.47) | <0.001 | 0.68(0.57,0.80) | <0.001 | 3.63(3.21,4.05) | <0.001 | 0.10(0.03,0.17) | 0.004 | 1.65(1.49,1.81) | <0.001 | 2.09(1.82,2.37) | <0.001 | 1.81(1.59,2.03) | <0.001 | 1.00(0.89,1.11) | <0.001 |
| Bulgaria | -1.25(-1.54,-0.96) | <0.001 | -2.20(-2.50,-1.90) | <0.001 | 0.62(0.44,0.79) | <0.001 | -0.51(-0.72,-0.30) | <0.001 | 0.12(0.01,0.24) | 0.036 | 1.82(1.31,2.33) | <0.001 | -0.08(-0.18,0.02) | 0.108 | 0.48(0.34,0.63) | <0.001 |
| Croatia | -1.99(-2.10,-1.89) | <0.001 | -1.92(-2.13,-1.71) | <0.001 | 0.98(0.88,1.08) | <0.001 | 0.50(0.37,0.63) | <0.001 | 0.94(0.86,1.01) | <0.001 | 1.23(1.04,1.42) | <0.001 | 1.35(1.26,1.45) | <0.001 | 0.82(0.75,0.89) | <0.001 |
| Czechia | -3.09(-3.21,-2.97) | <0.001 | -0.84(-1.14,-0.55) | <0.001 | 1.85(1.59,2.11) | <0.001 | -0.51(-0.77,-0.25) | <0.001 | 0.76(0.71,0.82) | <0.001 | -0.48(-0.69,-0.27) | <0.001 | 1.14(1.06,1.22) | <0.001 | 0.58(0.55,0.62) | <0.001 |
| Hungary | -2.39(-2.53,-2.25) | <0.001 | -1.88(-2.00,-1.76) | <0.001 | 1.26(1.03,1.48) | <0.001 | -0.93(-1.08,-0.77) | <0.001 | 0.59(0.55,0.63) | <0.001 | -0.49(-0.68,-0.30) | <0.001 | 0.61(0.57,0.64) | <0.001 | 0.09(0.07,0.11) | <0.001 |
| Montenegro | -0.14(-0.36,0.09) | 0.230 | 0.84(0.58,1.09) | <0.001 | 1.28(1.18,1.37) | <0.001 | 0.15(0.08,0.22) | <0.001 | 0.58(0.45,0.71) | <0.001 | 1.08(1.00,1.17) | <0.001 | 0.67(0.56,0.77) | <0.001 | 0.32(0.24,0.40) | <0.001 |
| Macedonia | -0.62(-0.81,-0.43) | <0.001 | -2.24(-2.50,-1.99) | <0.001 | 2.00(1.73,2.27) | <0.001 | -0.13(-0.17,-0.08) | <0.001 | 1.04(0.99,1.08) | <0.001 | 1.51(1.31,1.71) | <0.001 | 1.22(1.16,1.28) | <0.001 | 0.48(0.45,0.52) | <0.001 |
| Poland | -2.70(-2.83,-2.58) | <0.001 | -3.52(-3.87,-3.17) | <0.001 | 0.54(0.28,0.80) | <0.001 | -1.73(-1.97,-1.50) | <0.001 | 0.85(0.82,0.88) | <0.001 | 0.56(0.47,0.65) | <0.001 | 0.64(0.59,0.68) | <0.001 | 0.58(0.54,0.61) | <0.001 |
| Romania | -1.44(-1.77,-1.10) | <0.001 | -1.12(-1.26,-0.99) | <0.001 | 0.98(0.80,1.16) | <0.001 | -0.15(-0.29,-0.02) | 0.026 | 1.05(1.00,1.09) | <0.001 | 1.80(1.65,1.95) | <0.001 | 1.18(1.13,1.23) | <0.001 | 0.63(0.61,0.65) | <0.001 |
| Serbia | -1.68(-1.97,-1.39) | <0.001 | -1.94(-2.23,-1.66) | <0.001 | 1.04(0.94,1.14) | <0.001 | 0.31(0.22,0.40) | <0.001 | 0.91(0.83,1.00) | <0.001 | 1.20(1.07,1.33) | <0.001 | 0.89(0.78,0.99) | <0.001 | 0.40(0.32,0.49) | <0.001 |
| Slovakia | -2.14(-2.34,-1.94) | <0.001 | -0.67(-0.79,-0.55) | <0.001 | 0.03(-0.05,0.11) | 0.447 | -0.71(-0.79,-0.63) | <0.001 | 0.59(0.50,0.68) | <0.001 | 0.25(0.05,0.45) | 0.016 | 0.69(0.64,0.75) | <0.001 | 0.31(0.24,0.39) | <0.001 |
| Slovenia | -3.13(-3.30,-2.95) | <0.001 | -1.44(-1.57,-1.31) | <0.001 | -0.31(-0.63,0.00) | 0.051 | -0.27(-0.42,-0.12) | 0.001 | 0.87(0.80,0.94) | <0.001 | 0.29(0.02,0.55) | 0.034 | 0.82(0.73,0.90) | <0.001 | 0.46(0.43,0.50) | <0.001 |
| **Eastern Europe** |  |  |  |  |  |  |  |  |  |  |  |  |  |  |  |  |
| Belarus | 0.10(-0.40,0.60) | 0.691 | -3.96(-4.28,-3.63) | <0.001 | 0.15(-0.07,0.38) | 0.163 | -0.44(-0.55,-0.33) | <0.001 | 1.00(0.91,1.10) | <0.001 | 0.89(0.63,1.15) | <0.001 | 1.31(1.22,1.39) | <0.001 | 0.38(0.34,0.42) | <0.001 |
| Estonia | -2.09(-2.35,-1.83) | <0.001 | -2.76(-3.07,-2.45) | <0.001 | 2.29(1.91,2.67) | <0.001 | -0.98(-1.08,-0.88) | <0.001 | 1.29(1.23,1.35) | <0.001 | 1.18(0.98,1.39) | <0.001 | 1.91(1.82,2.01) | <0.001 | 0.78(0.74,0.82) | <0.001 |
| Latvia | -1.82(-2.23,-1.41) | <0.001 | -3.23(-3.60,-2.86) | <0.001 | 2.21(1.94,2.49) | <0.001 | -0.54(-0.69,-0.39) | <0.001 | 0.84(0.79,0.90) | <0.001 | 1.26(0.95,1.57) | <0.001 | 1.14(1.06,1.22) | <0.001 | 0.30(0.27,0.33) | <0.001 |
| Lithuania | -0.89(-1.26,-0.53) | <0.001 | -2.27(-2.57,-1.96) | <0.001 | 1.28(1.03,1.52) | <0.001 | 0.08(-0.09,0.25) | 0.36 | 0.69(0.65,0.73) | <0.001 | 1.15(0.85,1.46) | <0.001 | 1.28(1.22,1.34) | <0.001 | 0.52(0.49,0.55) | <0.001 |
| Moldova | -0.29(-0.61,0.04) | 0.082 | -2.44(-2.68,-2.19) | <0.001 | 1.02(0.85,1.19) | <0.001 | -0.67(-0.89,-0.44) | <0.001 | 1.37(1.18,1.56) | <0.001 | 1.45(1.11,1.80) | <0.001 | 1.30(1.16,1.45) | <0.001 | 0.98(0.84,1.12) | <0.001 |
| Russian Federation | -0.64(-1.30,0.03) | 0.060 | -4.36(-4.67,-4.05) | <0.001 | 1.13(0.93,1.33) | <0.001 | -0.90(-1.01,-0.78) | <0.001 | 1.17(1.09,1.24) | <0.001 | 0.64(0.40,0.88) | <0.001 | 1.39(1.32,1.46) | <0.001 | 0.54(0.52,0.56) | <0.001 |
| Ukraine | 0.34(-0.04,0.72) | 0.080 | -3.43(-3.87,-2.99) | <0.001 | 0.69(0.53,0.84) | <0.001 | -1.13(-1.27,-0.98) | <0.001 | 0.47(0.37,0.56) | <0.001 | 0.26(-0.03,0.54) | 0.074 | 1.01(0.94,1.07) | <0.001 | 0.16(0.10,0.23) | <0.001 |
| **Western Europe** |  |  |  |  |  |  |  |  |  |  |  |  |  |  |  |  |
| Cyprus | -2.47(-2.76,-2.18) | <0.001 | -0.44(-0.56,-0.31) | <0.001 | -1.11(-1.37,-0.84) | <0.001 | -0.49(-0.83,-0.16) | 0.005 | 1.09(0.93,1.26) | <0.001 | 0.98(0.75,1.21) | <0.001 | 0.77(0.67,0.86) | <0.001 | 0.83(0.73,0.93) | <0.001 |
| Greece | -1.30(-1.45,-1.15) | <0.001 | -0.27(-0.37,-0.17) | <0.001 | 0.83(0.75,0.91) | <0.001 | 1.02(0.85,1.19) | <0.001 | 0.62(0.44,0.80) | <0.001 | 0.57(0.41,0.74) | <0.001 | 0.70(0.63,0.78) | <0.001 | 0.49(0.43,0.54) | <0.001 |
| Israel | -4.36(-4.67,-4.04) | <0.001 | -1.82(-2.06,-1.59) | <0.001 | 0.34(-0.20,0.88) | 0.209 | 0.38(0.16,0.61) | 0.001 | 0.65(0.57,0.73) | <0.001 | -0.34(-0.60,-0.08) | 0.014 | 0.72(0.66,0.79) | <0.001 | 0.55(0.50,0.60) | <0.001 |

(DALYs, disability-adjusted life-years; BMI, Body Mass Index; BRI, Belt and Road Initiative.)
